# Sequelae of Foodborne Illness Caused by 5 Pathogens, Australia, Circa 2010

**DOI:** 10.3201/eid2011.131316

**Published:** 2014-11

**Authors:** Laura Ford, Martyn Kirk, Kathryn Glass, Gillian Hall

**Affiliations:** Australian National University, Canberra, Australian Capital Territory, Australia

**Keywords:** foodborne illness, Guillain-Barré syndrome, hemolytic uremic syndrome, irritable bowel syndrome, reactive arthritis, campylobacter, Salmonella, Australia, bacteria, Escherichia coli, Shiga toxin–producing E. coli, Campylobacter, Yersinia enterocolitica

## Abstract

In Australia circa 2010, 4.1 million (90% credible interval [CrI] 2.3–6.4 million) episodes of foodborne gastroenteritis occurred, many of which might have resulted in sequelae. We estimated the number of illnesses, hospitalizations, and deaths from Guillain-Barré syndrome, hemolytic uremic syndrome, irritable bowel syndrome, and reactive arthritis that were associated with contaminated food in Australia. Data from published studies, hospital records, and mortality reports were combined with multipliers to adjust for different transmission routes. We used Monte Carlo simulation to estimate median estimates and 90% CrIs. In Australia, circa 2010, we estimated that 35,840 (90% CrI 25,000–54,000) illnesses, 1,080 (90% CrI 700–1,600) hospitalizations, and 10 (90% CrI 5–14) deaths occurred from foodborne gastroenteritis–associated sequelae. *Campylobacter* spp. infection was responsible for 80% of incident cases. Reducing the incidence of campylobacteriosis and other foodborne diseases would minimize the health effects of sequelae.

## Abstract 2

Foodborne gastroenteritis results in a substantial amount of severe and disabling sequelae.

Foodborne gastroenteritis is a major source of illness in Australia, causing an estimated 4.1 million (90% credible interval [CrI] 2.3–6.4 million) illnesses, 30,600 (90% CrI 28,000–34,000) hospitalizations, and 60 (90% CrI 53–63) deaths each year (*1*). In addition to the direct effects of these illnesses, infection with some pathogens can result in sequelae, which can be severe, require multiple hospitalizations, and be costly to society (*2*). We report on the effects of sequelae associated with Guillain-Barré syndrome (GBS), hemolytic uremic syndrome (HUS), irritable bowel syndrome (IBS), and reactive arthritis (ReA) from 5 pathogens acquired from contaminated food in Australia.

Each of these 4 sequel illnesses are preceded by different gastrointestinal infections and have unique characteristics. GBS, a rare but serious autoimmune illness, affects the nervous system and causes acute flaccid paralysis. GBS can occur as a sequel to *Campylobacter* spp. infection 10 days–3 weeks after gastrointestinal illness (*3*,*4*). HUS is characterized by acute renal failure, hemolytic anemia, and thrombocytopenia and can result from infection with Shiga toxin–producing *Escherichia coli* (STEC) ≈4–10 days after onset of gastroenteritis (*5*,*6*). IBS is a gastrointestinal disorder that causes abdominal pain and bowel dysfunction. It is not life threatening, but it can cause substantial health effects after illness with *Campylobacter* spp., nontyphoidal *Salmonella enterica* serotypes (hereafter referred to as nontyphoidal *Salmonella* spp.), or *Shigella* spp. (*7*,*8*). ReA is a type of spondyloarthritis that can develop up to 4 weeks after an enteric infection from *Campylobacter* spp., nontyphoidal *Salmonella* spp., *Shigella* spp., or *Yersinia enterocolitica* (*9*). We estimated the number of illnesses, hospitalizations, and deaths resulting from GBS, HUS, IBS, and ReA from selected foodborne pathogens in Australia in a typical year circa 2010.

## Methods

We estimated the effects of foodborne sequelae acquired in Australia circa 2010 using data from multiple sources in Australia and from international peer-reviewed literature. We defined foodborne sequelae as illnesses occurring after bacterial gastroenteritis caused by eating contaminated food. Sequelae were defined as the secondary adverse health outcomes resulting from a previous infection by a microbial pathogen and clearly distinguishable from the initial health event (*10*). Illness can be acute, such as with HUS, or chronic (lasting for many years), as with IBS. We estimated incidence, hospitalizations, and deaths with uncertainty bounds using Monte Carlo simulation in @Risk version 6 (http://www.palisade.com/), which incorporates uncertainty in both data and inputs. Each stage of our calculation was represented by a probability distribution, and our final estimates of incidence, hospitalizations, and deaths were summarized by the median and 90% CrI. Similar to a recent study in the United States (*11*), we used empirical distributions for source distributions, such as the number of hospitalizations or deaths, to avoid assumptions about the expected shape of these distributions. All other inputs were modeled by using the PERT (project evaluation and review technique) distribution, which enables the input of a minimum, maximum, and modal value, or 3 percentile points, such as a median value and 95% bounds. We used this distribution widely in our analyses because it enables asymmetric distributions and can be produced from many data sources, including expert elicitation data. The Australian National University Human Research Ethics Committee approved the study.

### Incidence of Sequelae

Several pathogens are associated with the development of sequelae. Community estimates of foodborne illness from Kirk et al. (*1*) for *Campylobacter* spp., nontyphoidal *Salmonella* spp., *Shigella* spp., STEC, and *Y. enterocolitica* were used for estimating the incidence of foodborne sequelae (Table 1). Although *Shigella* spp. and nontyphoidal *Salmonella* spp. have been associated with HUS and STEC has been associated with IBS and ReA, data on which to base estimates are limited. In addition, although other pathogens, such as *Chlamydia trachomatis*, *Clostridium difficile*, *Giardia lamblia*, and norovirus, have been associated with these sequelae (*12*–*15*), we assessed only pathogens commonly associated with sequelae, domestically acquired, and with a foodborne transmission pathway. A “sequelae multiplier,” which is the proportion of sequelae cases that develop after enteric infection with a specific bacterial pathogen, was applied to our estimates of domestically acquired foodborne gastroenteritis cases caused by that pathogen (*1*). For each sequel illness, we reviewed relevant studies published during 1995–2012 using systematic reviews and studies using Australian data where possible to estimate the relevant sequelae multipliers. We reviewed articles about sequelae after infection with *Campylobacter* spp., *E. coli*, nontyphoidal *Salmonella* spp., *Shigella* spp., and *Y. enterocolitica*, and we estimated sequelae multipliers for GBS, HUS, IBS, and ReA after bacterial gastrointestinal infection on the basis of these reviews (Table 2). Relevant articles and additional information are documented in Technical Appendix 1.

**Table 1:** Pathogens associated with GBS, HUS, IBS, and ReA included in this study, Australia, circa 2010*

Pathogen	GBS	HUS	IBS	ReA
*Campylobacter* spp.	X		X	X
Nontyphoidal *Salmonella* spp.†			X	X
*Shigella* spp.			X	X
Shiga toxin–producing *Escherichia coli*		X		
*Yersinia enterocolitica*				X

*GBS, Guillain-Barré syndrome; HUS, hemolytic uremic syndrome; IBS, irritable bowel syndrome; ReA, reactive arthritis. †Nontyphoidal *S. enterica* serotypes.

**Table 2:** Sequelae multipliers extracted from the literature about domestically acquired foodborne bacterial gastroenteriti*

**Sequelae**	**ICD-10-AM code**	**Incidence after bacterial infection, %**
**GBS, median (range)**	G61.0	0.0304 (0.0192–0.0945)
**HUS, median (95% CI)**	D59.3	3 (1.7–5.1)
**IBS, median (90% CrI)**	K58.0	8.8 (7.2–10.4)
	K58.9	
**ReA, median (range)**	M02.1	7–12 (0–26)
	M02.3	
	M02.8	
	M03.2	

*CrI. credible interval; GBS, Guillain-Barré syndrome; HUS, hemolytic uremic syndrome; IBS, irritable bowel syndrome; ICD-10-AM, International Classification of Diseases, Tenth Revision, Australian Modification; ReA, reactive arthritis.

Our sequelae multiplier for GBS was based on 30.4 (range 19.2–94.5) cases of GBS per 100,000 cases of campylobacteriosis using data from studies from the United Kingdom, Sweden, and the United States (*16*–*18*). For HUS, the sequelae multiplier used was 3% (95% CI 1.7%–5.4%) from a South Australian study on STEC and HUS notifications during 1997–2009 (*19*). On the basis of data from Haagsma et al. (*20*), we assumed that 8.8% (95% CI 7.2%–10.4%) of foodborne disease caused by *Campylobacter* spp., nontyphoidal *Salmonella* spp., and *Shigella* spp. result in IBS. We used a separate sequelae multiplier for each pathogen that resulted in ReA. We assumed that 7% (range 2.8%–16%) of foodborne cases of *Campylobacter* spp., 8.5% (range 0%–26%) of foodborne cases of nontyphoidal *Salmonella* spp., 9.7% (range 1.2%–9.8%) of foodborne cases of *Shigella* spp., and 12% (range 0%–23.1%) of foodborne cases of *Y. enterocolitica* result in ReA (see full reference list in Technical Appendix 1). Total foodborne IBS and ReA cases reflect the sum of modeled IBS and ReA cases from these 3 and 4 pathogens, respectively. Details on the sequelae multipliers and incidence estimation methods are in online Technical Appendix 1 and Technical Appendix 2.

We compared the incidence of sequelae circa 2010 to that of sequelae circa 2000 by applying the same sequelae multipliers to estimates of the incidence of acute gastroenteritis to specific pathogens in 2006–2010 and 1996–2000, respectively. The estimates of incidence of acute gastroenteritis were based on notification data for *Campylobacter* spp., nontyphoidal *Salmonella* spp., *Shigella* spp., STEC, and *Y. enterocolitica* (*19*,*21*,*22*), (Technical Appendix 3).

### Hospitalizations and Deaths

To estimate hospitalizations associated with IBS from foodborne *Campylobacter* spp., nontyphoidal *Salmonella* spp., and *Shigella* spp. and hospitalizations associated with ReA from foodborne *Campylobacter* spp., nontyphoidal *Salmonella* spp., *Shigella* spp., and *Y. enterocolitica*, we used hospitalization data for 2006–2010 from all Australian states and territories, according to the International Classification of Diseases, Tenth Revision, Australian Modification (ICD-10-AM) codes. To estimate deaths for all 4 sequelae illnesses resulting from the respective foodborne pathogens, we used national death data for 2001–2010 from the Australian Bureau of Statistics, using ICD-10-AM codes (Technical Appendix 4). Principal diagnosis and additional diagnoses were included for hospitalizations, and underlying and contributing causes were included for deaths. Because we had only 1 year of hospitalization data for Victoria and 2 years for New South Wales, we extrapolated from these data to derive a distribution of the number of hospitalizations across all states, which was modeled as an empirical distribution. For these states, we assumed the same number of hospitalizations each year to adjust for missing data. Because of the severity of GBS and HUS, hospitalization estimates for these illnesses were not modeled, and all persons with estimated incident cases from contaminated food were considered to have been hospitalized.

We estimated incidences of hospitalization and death using a statistical model that incorporates uncertainty in case numbers and in multipliers using probability distributions (Figure), which is adjusted from the hospitalization estimation flow chart in Kirk et al. (*1*). We assumed that all estimated incident foodborne *Campylobacter*-associated GBS and STEC-associated HUS case-patients were hospitalized, so those cases were not modeled; however, multipliers were still needed for GBS and HUS to estimate deaths. Sequelae-associated deaths were estimated by using the same methods as for hospitalizations (Figure). Input data arose from the data sources discussed above or from multipliers that are discussed below.

**Figure:**
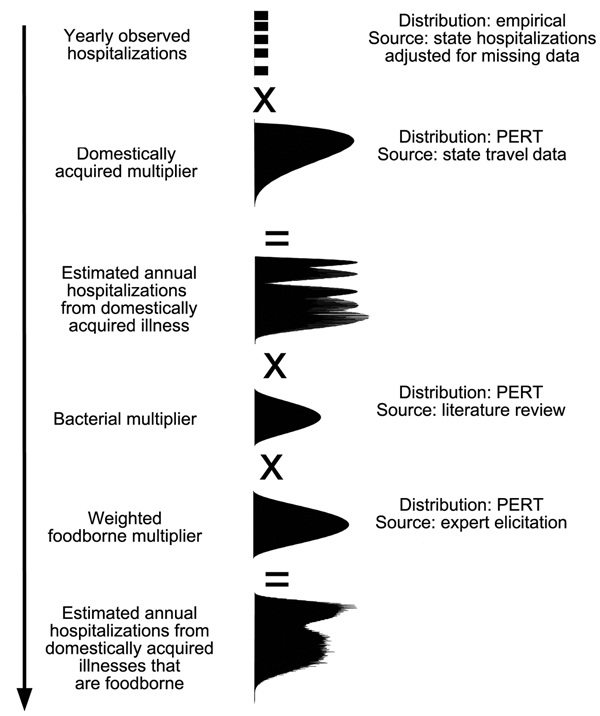
Flow chart for the approach used to calculate the estimated annual number of hospitalizations for sequelae associated with foodborne illness caused by 5 pathogens, Australia, circa 2010.

#### Domestically Acquired Multiplier

The “domestically acquired multiplier” adjusted for the proportion of case-patients who acquired their infection in Australia. We estimated domestically applied multipliers for the antecedent bacterial gastrointestinal pathogens using notifiable surveillance data from each state, extrapolated to give national estimates (*1*). We adopted the domestically acquired multiplier for *Campylobacter* spp. of 0.97 (90% CrI 0.91–0.99) for GBS and the domestically acquired multiplier for STEC 0.79 (90% CrI 0.73–0.83) for HUS (*1*). For IBS and ReA, a combined domestically acquired multiplier for *Campylobacter* spp., nontyphoidal *Salmonella* spp., and *Shigella* spp. for IBS and *Campylobacter* spp., nontyphoidal *Salmonella* spp., *Shigella* spp. and *Y. enterocolitica* for ReA was calculated as a weighted average of the domestically acquired multipliers for each pathogen, weighted by the total number of IBS and ReA cases for each pathogen, respectively (Technical Appendix 4; Technical Appendix 5).

#### Proportion Foodborne Multiplier

For each of the 4 sequelae, we calculated the proportion of hospitalizations and deaths from foodborne pathogens using 2 multipliers: a “bacterial multiplier” to attribute the proportion of overall cases of each of the sequelae illnesses to specific pathogens and a “foodborne multiplier” to attribute illnesses to foodborne exposure. The bacterial multiplier, which was the proportion of sequel cases attributable to their antecedent bacterial pathogen, was extracted from systematic reviews for GBS and HUS (*4*,*23*) and multiplied by the foodborne proportion for *Campylobacter* spp. and STEC, respectively. For IBS and ReA, from the literature we extracted a midpoint and range of the proportion of cases that resulted from infectious gastroenteritis (*12*,*20*,*24*). The IBS bacterial multiplier was then further multiplied by a foodborne multiplier for *Campylobacter* spp., nontyphoidal *Salmonella* spp., and *Shigella* spp., which was calculated as a weighted average of the foodborne multipliers for each pathogen, weighted by the total number of IBS cases for each pathogen. The ReA bacterial multiplier was then also multiplied by the foodborne multiplier for *Campylobacter* spp., nontyphoidal *Salmonella* spp., *Shigella* spp., and *Y. enterocolitica* by using a weighted average of the foodborne multipliers for each pathogen as was done for IBS (Technical Appendices 4 and 5).

## Results

### Incidence

We estimated that, circa 2010 in Australia, 70 (90% CrI 30–150) new cases of *Campylobacter*-associated GBS, 70 (90% CrI 25–200) new cases of STEC-associated HUS, 19,500 (90% CrI 12,500–30,700) new cases of *Campylobacter*-, nontyphoidal *Salmonella*– and *Shigella*-associated IBS, and 16,200 (90% CrI 8,750–30,450) new cases of *Campylobacter*-, nontyphoidal *Salmonella*-, *Shigella*-, and *Y. enterocolitica*–associated ReA were domestically acquired and caused by contaminated food (Table 3). We estimated that 35,840 (90% CrI 25,000–54,000) domestically acquired sequel illnesses resulted from foodborne gastroenteritis—an incidence rate of 1,620 (90% CrI 1,150–2,450) sequelae cases per million population. *Campylobacter* spp. infection resulted in the largest number of sequelae cases annually; ≈80% of the 36,000 sequel illnesses were attributable to *Campylobacter* spp. alone.

**Table 3:** Estimated number of sequelae illnesses resulting from domestically acquired foodborne bacterial gastroenteritis, Australia, circa 2010*

**Sequelae, pathogen**	**Median no. Illnesses (90% CrI)**	**Median rate (90% CrI)†**
**GBS, *Campylobacter* spp.**	70 (30–150)	3.1 (2–6)
**HUS, STEC**	70 (25–200)	3.3 (1–9)
**IBS**		
***Campylobacter* spp**	15,600 (9,000–26,500)	915 (570–1,440)
** Nontyphoidal *Salmonella* spp.‡**	3,500 (1,900–6,500)	
***Shigella* spp.**	30 (10–80)	
** Total**§	19,500 (12,500–30,700)	
**ReA**		
***Campylobacter* spp.**	12,500 (5,500–25,500)	765 (415–1,375)
** Nontyphoidal *Salmonella* spp.‡**	3,250 (700–9,000)	
***Shigella* spp.**	29 (10–75)	
* Yersinia enterocolitica*	150 (50–300)	
** Total**§	16,200 (8,500–30,000)	
**Total**	35,840 (25,000–54,000)	1,620 (1,150–2,450)

*CrI, credible interval; GBS, Guillain-Barré syndrome; HUS, hemolytic uremic syndrome; IBS, irritable bowel syndrome; ReA, reactive arthritis; STEC, Shiga toxin–producing *Escherichia coli*. †No. cases per million population. ‡i.e., nontyphoidal *S. enterica* serotypes. **§Simulated values, which might not add to total because of rounding and variation over simulations.**

#### Comparison with Estimates Circa 2000

Using data circa 2000, we estimated that 50 GBS cases, 55 HUS cases, 14,800 IBS cases, and 12,500 ReA cases occurred each year. Elsewhere, we estimated that the rate of foodborne campylobacteriosis was approximately 13% higher in 2010 than 2000 (*1*); this increase led to a 13% increase in *Campylobacter*-associated GBS in 2010 over 2000. Similarly, we estimated that the rate of foodborne salmonellosis was 24% higher in 2010 than in 2000 (*1*). These factors combine to explain much of the increase in IBS and ReA. The rate of STEC-associated HUS remained about the same in 2000 and 2010 (Technical Appendix 3).

### Hospitalizations and Deaths

We estimated that, circa 2010 in Australia, 1,080 (90% CrI 700–1,600) hospitalizations for sequelae illnesses occurred from domestically acquired foodborne gastroenteritis, equating to 50 (90% CrI 30–70) hospitalizations per million population per year (Table 4). We estimated a total of 10 (90% CrI 5–14) deaths from sequelae to domestically acquired foodborne gastroenteritis—a rate of 0.5 (90% CrI 0.2–0.6) deaths per million population per year (Table 4).

**Table 4:** Estimated number of sequelae-associated hospitalizations and deaths caused by domestically acquired foodborne bacterial gastroenteritis, Australia, circa 2010*

**Sequelae**	**Hospitalizations**			**Deaths**	
	Median no. (90% CrI)	Rate (90% CrI)†		Median no. (90% CrI)	Rate (90% CrI)†
**GBS**	70 (30–150)	3.1 (2–6)		6 (2–10)	0.3 (0.1–0.5)
**HUS**	70 (25–200)	3.3 (1–9)		2 (1–3)	0.1 (0.03–0.12)
**IBS**	915 (550–1,400)	43 (25–70)		2 (1–2)	0.1 (0.05–0.11)
**ReA**	25 (20–40)	1 (1–2)		0	0
**Total**	1,080 (700–1,600)	50 (30–70)		10 (5–14)	0.5 (0.2–0.6)

*CrI, credible interval. GBS, Guillain-Barré syndrome; HUS, hemolytic uremic syndrome; IBS, irritable bowel syndrome; ReA, reactive arthritis; STEC, Shiga toxin–producing *Escherichia coli*. **†Cases per million population.**

## Discussion

Our study demonstrates that foodborne gastroenteritis in Australia results in substantial severe and disabling sequelae. We estimated a yearly rate of 1,620 incident cases of sequelae illnesses, 50 hospitalizations, and 0.5 deaths per million population circa 2010. In addition, a comparison with estimates recalculated for 2000 indicates an increase in the rates of GBS, IBS, and ReA since 2000, which is consistent with and directly related to rising levels of antecedent foodborne illnesses caused by *Campylobacter* spp. and nontyphoidal *Salmonella* spp. during this period (*1*). This increase highlights the importance of quantifying sequelae when estimating the effects of foodborne disease and provides further impetus for reducing illness from foodborne bacterial pathogens.

The impact of *Campylobacter* spp. infection in the community is high. Approximately 179,000 cases of foodborne campylobacteriosis occur in Australia each year (*1*), and *Campylobacter* spp. was responsible for 80% of the foodborne sequelae illness estimated in this study. The reported rate of infection from *Campylobacter* spp. in Australia has increased since 2010 (*1*) and is higher than in many other industrialized countries. For example, the rate of *Campylobacter* spp. for Australia was ≈10 times higher than that for the United States (*25*), double that for the Netherlands (*26*), and slightly higher than that for the United Kingdom (*27*). In the Netherlands, a lower rate of acute *Campylobacter* spp. gastroenteritis has contributed to lower estimates of rates of sequel illnesses than our estimates for GBS, IBS, and ReA (*26*).

In New Zealand, food safety interventions have been effective in lowering campylobacteriosis rates and sequelae. In 2006, high campylobacteriosis notification rates (>3,800 cases per million population) prompted increased research on *Campylobacter* spp., which resulted in the introduction of food safety and poultry industry interventions, including *Campylobacter* spp. performance targets at primary processing plants and promotion of freezing all fresh poultry meat (*28*). By 2008, the rate of campylobacteriosis notifications decreased by 54% to 1,615 cases per million population (*28*). In addition, after these interventions in New Zealand, the rate of GBS hospitalizations decreased by 13% (*29*). The less dramatic decrease in GBS than in campylobacteriosis might be explained by the fact that *Campylobacter* spp. is not the only cause of GBS. If Australia were to experience decreases similar to those in New Zealand, we would expect the rate of foodborne campylobacteriosis in the community to drop from approximately 8,400 to 3,864 cases per million population. Sequelae would decrease from 1,620 to 870 cases per million population per year. Furthermore, total GBS-associated hospitalizations, including GBS from all causes and readmissions, would decrease from ≈73 to 63 hospitalizations per million population annually.

A comparison of our foodborne *Campylobacter*-associated GBS incidence estimates with raw hospitalization data showed many more hospitalizations than incident cases. This finding probably is attributable to repeat hospitalizations. We took a conservative approach by basing incidence estimates on community estimates of campylobacteriosis and assuming that all persons with incident cases were hospitalized. A yearly median of 1,536 (range 1,428–1,632) primary and additional GBS diagnoses occurred in Australian hospitals during 2006–2010 (including GBS from all causes and readmissions) and equates to a median rate of 73.1 (range 64.7–77.4) GBS-associated hospitalizations per million population each year. This rate is within the range from a New Zealand study, which found a median rate of 56.3 (range 42.1–75.9) GBS-associated hospitalizations during a 13-year period, with ≈41% of case-patients being readmitted, resulting in 23.2 (range 15.3–29.3) incident GBS hospitalizations per million population each year (*29*). If we assume that 41% of Australia’s 1,536 GBS hospitalizations are readmissions and apply the domestically acquired multiplier and foodborne proportion multiplier used to estimate GBS-associated deaths (Technical Appendix 4), we would estimate 170 (90% CrI 60–265) incident foodborne *Campylobacter*-associated GBS hospitalizations. This point estimate is higher than our current estimate of 70, although the credible interval includes our estimate. A validation study of medical records of persons with GBS would enable us to better characterize readmissions for GBS.

Our approach has several limitations. First, our comparison of sequelae estimates for 2000–2010 assumes a constant rate of sequelae illness after gastrointestinal infection over time. Although our methods provide an indirect method of assessing changes in sequelae incidence over time, the approach is useful because it enables comparison of the population-level effect of sequelae at these 2 time points. Second, our study measured incidence and not prevalence of sequelae. We estimated the number of new cases every year and did not quantify the long-term effects of these sequelae. Third, our study does not estimate all sequelae illness from foodborne disease pathogens. We did not include sequelae, such as end-stage renal disease, inflammatory bowel disease, and encephalitis, in our estimates. We chose GBS, HUS, IBS, and ReA for this study because they were known, well studied, and well characterized in available data sources. These provide a good basis to begin to understand the effects of foodborne sequelae and the policy implications of reducing illness from preceding bacterial pathogens.

Our estimates for GBS, HUS, IBS, and ReA incidence relied heavily on the quality of the literature we reviewed. We used Australian data and systematic reviews wherever possible. The Australian hospitalization and deaths data we used were of high quality and included both principal and additional diagnoses from all states. However, because data were missing from some states in some years, we extrapolated from these data to the remaining years. Finally, ICD-10 and ICD-10-AM coding can be problematic when co-morbid conditions are present, when hospital transfers occur, or when diagnostic criteria are inconsistent. Therefore, our estimates for sequelae hospitalizations and deaths may be conservative because they do not account for these coding errors.

The sequelae estimates from this study showed that the impact of foodborne *Campylobacter* spp., nontyphoidal *Salmonella* spp., *Shigella* spp., STEC, and *Y. enterocolitica* was much greater then when consideration is given simply to the initial acute illness. *Campylobacter* spp. infection, in particular, was highlighted as an increasing problem in Australia. Our estimates provide a basis for costing studies, which can be useful for developing food safety policies and interventions. Finally, our study highlights the need for better data from large population-based studies in Australia to further characterize sequelae, as well as foodborne pathogens.

Technical Appendix 1 Sequelae incidence after bacterial gastroenteritis: the sequelae multiplier.

Technical Appendix 2 Methods to estimate sequelae incidence.

Technical Appendix 3 Comparison with estimates from 2000.

Technical Appendix 4 Methods to estimate sequelae hospitalizations and deaths.

Technical Appendix 5 Model inputs for 4 sequelae illnesses due to contaminated food.
