# Supplementary material for: Sequelae of Foodborne Illness Caused by 5 Pathogens, Australia, Circa 2010
Source: Emerg Infect Dis. 2014 Nov;20(11):1865–71. doi: 10.3201/eid2011.131316 (PMC4214289; doi:10.3201/eid2011.131316)
Supplement: Technical Appendix 1 — Sequelae incidence after bacterial gastroenteritis: the sequelae multiplier. [file 13-1316-Techapp-s1.pdf]

Article DOI: http://dx.doi.org/10.3201/eid2011.131316

Sequelae of Foodborne Illness Caused by 5 Pathogens, Australia, Circa 2010

Technical Appendix 1

Sequelae Incidence after Bacterial Gastroenteritis: The Sequelae Multiplier

For each sequel, a multiplier was used that estimated the proportion of bacterial gastroenteritis cases that developed into chronic sequelae. This appendix summarizes the relevant studies published during 1995–2012, which we selected for review, as well as the sequelae multipliers that were estimated for Guillain-Barré syndrome (GBS), hemolytic uremic syndrome (HUS), irritable bowel syndrome (IBS), and reactive arthritis (ReA).

GBS

A few studies have quantified the incidence of GBS illness following *Campylobacter* spp. infection by using large cohorts of patients or the literature (online Technical Appendix 1 Table 1). In a population-based cohort study in the United Kingdom, including 2 months of follow-up, 3 cases of GBS occurred among 15,587 *Campylobacter* spp. cases. This yielded a rate of 19.2 cases of GBS per 100,000 cases of campylobacteriosis (*1*). In Sweden, 0.03% of a cohort of 29,567 persons with laboratory-confirmed *C. jejuni* infection developed GBS illness after 2 months of follow-up, yielding an annual incidence of 30.4 cases of GBS per 100,000 cases (95% CI 13.9–57.8) of *C. jejuni* infection (*2*). In a literature review, Allos (*3*) estimated that in the United States, GBS develops in 1 of every 1,058 cases, or 94.5 per 100,000 cases, of *C. jejuni* infection. Baker et al. (*4*) performed a study of hospital records in New Zealand, which found a rate of 414 cases of GBS per 100,000 *Campylobacter* spp. hospitalizations.

For the sequelae multiplier, a midpoint of 30.4 cases of GBS per 1000,000 cases of campylobacteriosis was taken from the study by McCarthy and Gieseke (*2*) using a minimum value of 19.2 per 100,000 from the UK study and a maximum value of 94.5 per 100,000 from the study by Allos (*3*). Although the study by Baker et al. (*4*) is a valuable one, we excluded it from

the calculation of our sequelae multiplier because persons hospitalized with *Campylobacter* spp. infection may not be representative of *Campylobacter* spp. cases in the community.

Technical Appendix 1 Table 1. Incidence of GBS after infection with *Campylobacter* spp.*

Reference	Study years	Type of study	Country	No. GBS cases/*Campylobacter* spp. patients	Incidence per 100,000 (95% CI)
Baker et al. (*4*)	1995–2008	Cohort	New Zealand	35/8,448 hospitalizations	414 (373–459)
Tam et al. (*1*)	1991–2001	Cohort	UK	3/15,587 cases	19.2 (17.1–21.5)
McCarthy and Giesecke (*2*)	1987–1995	Cohort	Sweden	9/29,563 cases	30.4 (13.9–57.8)
Allos (*3*)	1964–1996†	Review and estimation	Global/USA	1/1058 cases	94.5 (2.4–525)

*GBS, Guillain-Barré syndrome.
†Years of reviewed studies.

HUS

A variety of organisms, drugs and conditions can initiate the symptoms of HUS, but the majority of HUS cases are post-diarrheal—usually caused by Shiga toxin–producing *Escherichia coli* (STEC) (*5*). In developed communities, STEC is the most commonly implicated organism in HUS (*6*), and in children, 90% of HUS cases are due to STEC (*5*). HUS is also associated with *Shigella dysenteria* serotype 1, particularly in less developed communities (*6*); however, a recent systematic review was unable to find an adequate number of studies to quantify the association between *S. dysenteria* serotype 1 and HUS (*7*). In addition, in a few studies, HUS has been associated with *Clostridium difficile* and *Salmonella enterica* serotype Typhi, but the evidence is limited (*8–10*). Therefore we estimated food-related HUS cases as a sequel to STEC, which may create an underestimation of HUS if there are food-related HUS cases in Australia from other organisms.

Several sources have reported that 3%-7% of sporadic STEC infections develop into HUS (*11–14*). Australian studies support this estimate range. Vally et al. (*15*) examined South Australian surveillance data and identified 14 HUS cases and 460 STEC cases, resulting in an estimate of 3% of STEC cases developing into HUS. Sixty percent of HUS case-patients were $\leq$15 years of age. In addition, in a case–control study in 6 Australian jurisdictions, 113 STEC case-patients were identified, 44 of whom were infected with O157 and 66 who were infected with non-O157 (*14*). Eight (7%) of all the STEC cases, 1 (2%) case-patient with O157, and 7 (10%) case-patients infected with non-O157 developed HUS (*14*). Although STEC O157 is more commonly associated with HUS worldwide (*6*), data on geographic differences in STEC serotypes suggest that in Australia, “non-O157:H7 STEC strains predominate,” and STEC O157:H7 is not as frequently implicated in “diarrhea-associated HUS” (*16*).

Overseas studies have reported higher proportions of STEC infections developing into HUS. In a cohort study of Argentinian children, aged ≤15 years, 8 (8.6%) of 93 STEC patients developed HUS (*17*). Through enhanced surveillance in the Netherlands, Van Duynhoven et al. (*18*) found that HUS developed in 12 of 82 (14.6%) patients. Seventy-five percent of HUS case-patients were ≤15 years (*18*). With the highest proportion from all reviewed studies, a Swiss linkage study found that HUS developed in 13 (29.5%) of 44 STEC patients, all of whom were ≤15 years of age (*19*). Several studies on the incidence of HUS after STEC outbreaks have found that ≈20% of STEC cases develop into HUS (*20–23*). However, Sigmundsdottir et al. found no HUS cases among 9 STEC outbreak patients in Iceland (*24*) (Technical Appendix 1 Table 2).

A sequelae multiplier proportion of 3% (95% CI 1.7%–5.4%) was chosen, based on the South Australian study by Vally et al. (*15*). This study was chosen because STEC surveillance in South Australia is more complete than for other Australian states (*11*) and would therefore give a more representative estimate for Australia than the other available studies.

Technical Appendix 1 Table 2. Incidence of HUS after STEC*

Reference	Study years	Study type	Country	Age of HUS case-patients	No. HUS cases/no. STEC cases	STEC cases developing into HUS, %
Bradley et al. (*20*)	2008	Epidemiology investigation and case–control: after an outbreak	USA	Median: 46 y (range1–88 y), 60% adult	11/56	20
Lopez et al. (*17*)	2006	Prospective cohort	Argentina	≤15 y	8/93	8.6
Neil et al. (*21*)	2009	Case–control: after an outbreak	USA	Not stated	10/57	18
Vally et al. (*15*)	1997–2009	Surveillance	Australia	Range: <5–60+, 60% aged ≤15 y	14/460	3
Frank et al. (*22*)	2011	Surveillance: after an outbreak	Germany	Median: 42, 88% aged >15 y	845/3816	22
Kappelli et al. (*19*)	2000–2009	Linkage	Switzerland	Median: 3.5 y (range 0–15 y)	13/44	29.5
McPherson et al. (*14*)	2003–2007	Case–control	Australia	Median: 4 y (range 1–62)	8/113	7
Sigmundsdottir et al. (*24*)	2007	Cohort: after an outbreak	Iceland	Not stated	0/9	0
Rangel et al. (*25*)	1982–2002	Outbreak surveillance	USA	Not stated	354/8598	4.1
Jay et al. (*23*)	1999	Epidemiology investigation and case–control: after an outbreak	USA	Not stated	3/13	23
Van Duynhoven et al. (*18*)	1999–2001	Enhanced surveillance	The Netherlands	Range: 0–70 y, 75% aged ≤15 y	12/82	14.6

*HUS, hemolytic uremic syndrome; STEC, Shiga toxin–producing *Escherichia coli*.

IBS

There have been a few systematic reviews and/or meta-analyses on the association between intestinal infection and post-infectious IBS (PI-IBS). A recent review suggests the proportion of persons developing IBS following gastrointestinal infection is 4%–35% (*26*). In

2010, Haagsma et al. (*27*) found that 1 year after infection from nontyphoidal *S. enterica* serotypes (hereafter referred to as nontyphoidal *Salmonella* spp.), nontyphoidal *Salmonella* spp., *Shigella* spp., or *Campylobacter* spp., IBS developed in 9% (95% CI 7.2–10.7) of patients. Similarly, in a systematic review of 18 studies, Thabane et al. (*28*) found a pooled incidence of PI-IBS of 10% (95% CI 9.4–85.6). Comparably, Halvorson et al. (*29*) reviewed 8 studies on nontyphoidal *Salmonella* spp., *Shigella* spp., bacterial unspecified, or unspecified, and their association with IBS, and calculated a median prevalence of IBS of 9.8% (interquartile range 4.0–13.3) in the exposed group and 1.2% Interquartile rate range 0.04–1.8) in the control group. A review by Smith and Bayles (*30*) found a mean prevalence of PI-IBS of 15% from 15 studies, with species of *Campylobacter*, nontyphoidal *Salmonella* spp., and/or *Shigella* as the most common agents of infection.

In the United Kingdom, Neal et al. (*31*) performed a postal survey and found that 25% of subjects had persistently altered bowel habits after bacterial gastroenteritis from nontyphoidal *Salmonella* spp., *Shigella* spp., or *Campylobacter* spp.; however, only 7% met the Rome criteria for new IBS. Also in the United Kingdom, Parry et al. (*32*) looked at the relationship between IBS and bacterial gastroenteritis from *Campylobacter* spp., nontyphoidal *Salmonella* spp., *Shigella* spp., *E. coli* O157, and *Aeromonas sobria*, and calculated an incidence of new IBS of 16.7% in the exposed group and 1.9% in the control group.

Studies looking at singular pathogens have also found an association between infectious gastroenteritis outbreaks and IBS. After an outbreak in 2002 in Spain, Mearin et al. (*33*) noted that before the outbreak, the prevalence of IBS was similar in case-patients and controls (2.9% vs. 2.3%); however, 3 months after the outbreak, IBS prevalence in case-patients had increased (9.2% vs. 1.7%), and 12 months after the outbreak, prevalence in case-patients remained higher (10.2% vs. 0.7%). The cumulative incidence was 7.4% at 3 months, 10.9% at 6 months, and 11.6% at 12 months. In Korea, 12 months after a *Shigella* spp. outbreak, Ji et al. (*34*) found that IBS had developed in 15 (14.9%) of 101 case-patients and 6 of 102 (5.9%) controls. In Canada, 2–3 years after an outbreak of *E. coli* O157:H7 and *Campylobacter* spp., 27.5% of 904 subjects with self-reported gastroenteritis reported IBS, and 36.2% of 464 subjects with clinically suspected gastroenteritis reported IBS (*35*). In a pediatric cohort from the Canadian outbreak, the cumulative incidence of PI-IBS for exposed subjects was 10.5% vs. a cumulative incidence in controls of 2.5% (*36*).

There have been studies on the association of *G. lamblia* with IBS; however, these have produced inconsistent results. While Wensaas et al. (*37*) found a high prevalence of IBS in exposed patients 2 years after acute giardiasis, Penrose et al. (*38*) found no linear association between *G. lamblia* and IBS, and a study by D'Anchino et al. (*39*) concluded that *G. lamblia* infection is a trigger for exacerbating preexisting IBS but could not conclude that *G. lamblia* causes IBS. PI-IBS has also been shown to develop after norovirus. Marshall et al. (*40*) performed a 2-year study after a norovirus outbreak; of the 89 respondents who reported an acute enteric illness during the outbreak and did not have preexisting IBS, 23.6% reported symptoms consistent with PI-IBS at 3 months versus 3.4% who reported symptoms but remained well during the outbreak. However, at 6, 12, and 24 months, the prevalence of IBS did not differ statistically among exposed and unexposed individuals, suggesting that PI-IBS might be more transient after viral gastroenteritis than it is after bacterial dysentery (*40*) (Technical Appendix 1 Table 3).

The meta-analysis by Haagsma et al. (*27*), which suggests that IBS develops in ≈9% (95% CI 7.2%–10.7%) of *Campylobacter* spp., nontyphoidal *Salmonella* spp., and *Shigella* spp. case-patients at 10–12 months of follow-up was chosen as the sequelae multiplier to simulate the plausible proportion of these bacterial pathogens that cause IBS using an alternate PERT distribution. While studies of multiple pathogens have found different rates of PI-IBS depending on etiology, this proportion was chosen for all 3 pathogens because it is a pooled rate that comes from a recent meta-analysis and is similar to PI-IBS rates after bacterial gastroenteritis that were reported in other studies (*28*,*29*,*41*).

Technical Appendix 1 Table 3. Incidence of IBS after infection with enteric pathogens, Australia, circa 2010*

Reference	Year of publication	Study years	Country	Study type	Foodborne pathogen	IBS patients after infectious gastroenteritis, %
Koh et al. (*41*)	2012	2008–2010	Korea	Prospective cohort	Nontyphoidal *Salmonella* spp., *Shigella* spp., STEC O157, *Vibrio cholerae*	9.2% at 3 mo†, 12.3% at 6 mo†
Wensaas et al. (*37*)	2012	2007–2008	Norway	Historic cohort	*Giardia lamblia*	46.1% at 3 y
Schwille-Kiuntke et al. (*26*)	2011	-	Global	Systematic review	*Campylobacter* spp., *Escherichia coli*, *G. lamblia*, norovirus, nontyphoidal *Salmonella* spp, *Shigella* sp., *Trichinella britovi*; bacterial, viral, and parasitic gastroenteritis and travelers' diarrhea	4%–36% Incidence range
Thabane et al. (*36*)	2010	2002–2008	Canada	Outbreak study	*E. coli* O157:H7, *Campylobacter* spp.	10.5%†
Haagsma et al. (*26*)	2010	-	The Netherlands	Meta-analysis	*Campylobacter* spp., nontyphoidal *Salmonella* spp.,	9% (95% CI 7.2–10.7)

Reference	Year of publication	Study years	Country	Study type	Foodborne pathogen	IBS patients after infectious gastroenteritis, %
					Shigella spp.	at 1 y
Marshall et al. (*35*)	2009	2002–2008	Canada	Outbreak study	*E. coli* O157:H7, *Campylobacter* spp.	27.5% (self-reported), 36.2% (clinically suspected)
Thabane et al. (*28*)	2007	-	Canada, China, Israel, Korea, New Zealand, UK, USA	Systematic review and meta-analysis	*Campylobacter* spp., nontyphoidal *Salmonella* spp., *Shigella* spp., confirmed bacterial gastroenteritis, and self-reported illness	10% (95% CI 9.4–85.6), 4%–32% incidence range
Marshall et al. (*40*)	2007	2002–2004	Canada	Outbreak study	Norovirus	23.6% at 3 mo
Smith and Bayles (*30*)	2007	-	Canada, China, Korea, Spain, UK, USA	Systematic review	*Campylobacter* spp., Cryptosporidium spp., E.*coli*, *G. lamblia*, nontyphoidal *Salmonella* spp., *Shigella* spp.	15% (range 3.4–31.6)‡
Halvorson et al. (*29*)	2006	-	Canada, China, Korea, Spain, UK, USA	Systematic review and meta-analysis	Nontyphoidal *Salmonella* spp., *Shigella* spp., bacterial, and unspecified	9.8% (IQR 4.0–13.3)‡
Ji et al. (*34*)	2005	2001–2002	Korea	Outbreak study	*Shigella* spp.	14.9% at 1 y
Mearin et al. (*33*)	2005	2002–2003	Spain	Cohort study after an outbreak	Nontyphoidal *Salmonella* spp.	7.4% at 3 mo†, 10.9% at 6 mo†, 11.6% at 1 y†
Parry et al. (*32*)	2003	2000–2001	UK	Prospective case–control study	*Campylobacter* spp., nontyphoidal *Salmonella* spp., *Shigella* spp., STEC O157, *Aeromonas sobria*	16.7% at 6 mo
Neal et al. (*31*)	1997	1994	UK	Cross-sectional	*Campylobacter* spp., nontyphoidal *Salmonella* spp., and *Shigella* spp.	7% at 6 mo

*IBS, irritable bowel syndrome; IQR, interquartile range; nontyphoidal *Salmonella* spp., nontyphoidal *S. enterica* serotypes; STEC, Shiga toxin–producing *E. coli*.
†Cumulative incidence.
‡Median prevalence.

ReA

The causes of ReA are ambiguous because no formal definition or agreed-upon diagnostic criteria exist (*42*,*43*). Although the primary focus of the infection is usually through the gut or urogenital track, ReA has also been associated with respiratory pathogens (*42*). The classical gastrointestinal microbes resulting in ReA are *Yersinia enterocolitica,* nontyphoidal *Salmonella* spp., *Shigella* spp., and *Campylobacter* spp (*43*). and most agree that the term "ReA" should be applied only to infection caused by these gastrointestinal pathogens and *Chlamydia* spp (*43*); however, nonclassical ReA forms have been associated by a variety of other bacteria, including *Brucella* and *Staphylococcus,* and many authors have applied the term ReA for arthritis after infection with *C. difficile*, *Cryptosporidium*, *Giardia lamblia*, *E. coli*, and *Strongyloides* spp (*43*,*44*). With the majority of the literature focusing on the 4 classical gastrointestinal pathogens as triggers for ReA, we chose to use these to estimate the incidence of ReA due to contaminated food. If other enteric pathogens are in fact associated with ReA, our estimates of foodborne ReA may be conservative.

We were unable to find any published systematic reviews that report a global incidence rate for ReA after infection with the bacterial pathogens *Campylobacter* spp., nontyphoidal *Salmonella* spp., *Shigella* spp., and *Y. enterocolitica*. Because there are no diagnostic criteria for ReA, the case definition and the resulting incidences vary (*42*). The literature suggests that the incidence of ReA as a sequel to bacterial gastroenteritis varies by the enteric pathogen. For each of the bacterial enteric pathogens that precede ReA, we compiled papers that reported the proportion of cases that developed into ReA published in 2000 or later where all enteric cases were confirmed by a laboratory (Technical Appendix 1 Table 4). Because there is still quite a bit of variation in incidence in studies by pathogen, the median and range for *Campylobacter* spp., nontyphoidal *Salmonella* spp., *Shigella* spp., and *Y. enterocolitica* from the studies in Technical Appendix 1 Table 4 were calculated for the sequelae multiplier and used to simulate a distribution of the plausible proportion of cases that result in this sequel using an alternate PERT or PERT distribution, respectively. From the literature, we assume that 7% (range 2.8%-16%) of foodborne *Campylobacter* spp., 8.5% (range 0%-26%) of foodborne nontyphoidal *Salmonella* spp., 9.7% (range 1.2%-9.8%) of foodborne *Shigella* spp., and 12% (range 0%-23.1%) of foodborne *Y. enterocolitica* result in ReA. These distributions were then applied to the estimates of domestically acquired foodborne cases for each of the preceding bacterial pathogens.

Technical Appendix 1 Table 4. ReA incidence* by foodborne pathogen, Australia, 2010

Reference	Study years	Study type	Country	ReA cases/gastroenteritis cases
				ReA cases/*Campylobacter* spp. cases
Schonberg-Norio et al. (*45*)	2002	Cross sectional	Finland	8/201 (4.0%)
Doorduyn et al. (*46*)	2005	Case–control	The Netherlands	20/434 (4.6%)
Townes et al. (*47*)	2002–2004	Cohort	USA	302/2384 (12.7%)
Schiellerup et al. (*48*)	2002–2003	Case–case comparison	Denmark	131/1003 (13.1%)
Pope et al. (*49*)	1966–2006	Review	Europe	1%–5%
Rees et al. (*50*)	1998–1999	Cohort	USA	9/324 (2.8%)
Hannu (*51*)	1997–1998	Cohort	Finland	45/609 (7.4%)
Locht and Krogfelt (*52*)	1997–1999	Cohort	Denmark	27/173 (15.6%)
				ReA cases/nontyphoidal *Salmonella* spp. cases
Arnedo-Pena et al. (*53*)	2005	Outbreak study	Spain	6/67 (9%)
Doorduyn et al. (*46*)	2005	Case–control	The Netherlands	8/181 (4.4%)
Townes et al. (47)	2002–2004	Cohort	USA	204/1356 (15.0%)
Schiellerup et al. (*48*)	2002–2003	Case–case comparison	Denmark	104/619 (16.8%)
Lee et al. (*54*)	1999	Outbreak study	Australia	38/261 (14.6%)
Rees et al. (*50*)	1998–1999	Cohort	USA	2/100 (2.0%)
Buxton et al. (*55*)	1999–2000	Case–control	Canada	17/66 (25.7%)
Hannu et al. (*56*)	1999	Outbreak study	Finland	5/63 (7.9%)
Rudwaleit et al. (*57*)	1998	Outbreak study	Germany	0/286 (0%) (children only)
Urfer et al. (*58*)	1993	Outbreak study	Switzerland	1/156 (0.6%)
				ReA cases/*Shigella* spp. cases
Townes et al. (*47*)	2002–2004	Cohort	USA	29/298 (9.7%)
Schiellerup et al. (*48*)	2002–2003	Case–case comparison	Denmark	10/102 (9.8%)
Rees et al. (*50*)	1998–1999	Cohort	USA	1/81 (1.2%)

Reference	Study years	Study type	Country	ReA cases/gastroenteritis cases
				ReA cases/*Yersinia enterocolitica* cases
Huovinen et al. (*59*)	2006	Case–control	Finland	11/248 (4.4%)
Townes et al. (*47*)	2002–2004	Cohort	USA	5/35 (14.3%)
Schiellerup et al. (*48*)	2002–2003	Case–case comparison	Denmark	21/91 (23.1%)
Rees et al. (*50*)	1998–1999	Cohort	USA	0/8 (0%)
Hannu et al. (*60*)	1998	Outbreak study	Finland	4/33 (12.1%)

*Incidence of ReA after *Campylobacter* spp. infection: median 7%, range 2.8%–16%; after *Salmonella* spp. infection: median 8.5%, range 0%–26%; after *Shigella* spp. infection: median 9.7%, range 1.2%–9.8%; after *Yersinia enterocolitica* infection: median 12%, range 0%–23.1%. ReA, reactive arthritis. Nontyphoidal *Salmonella* spp., nontyphoidal *S. enterica* serotypes.

References

1. Tam CC, Rodrigues LC, Petersen I, Islam A, Hayward A, O'Brien SJ. Incidence of Guillain-Barré syndrome among patients with *Campylobacter* infection: a general practice research database study. J Infect Dis. 2006;194:95–7. PubMed http://dx.doi.org/10.1086/504294

2. McCarthy N, Giesecke J. Incidence of Guillain-Barré syndrome following infection with *Campylobacter jejuni*. Am J Epidemiol. 2001;153:610–4. PubMed http://dx.doi.org/10.1093/aje/153.6.610

3. Allos BM. Association between *Campylobacter* infection and Guillain-Barré syndrome. J Infect Dis. 1997;176(Suppl 2):S125–8. PubMed http://dx.doi.org/10.1086/513783

4. Baker MG, Kvalsvig A, Zhang J, Lake R, Sears A, Wilson N. Declining Guillain-Barré syndrome after campylobacteriosis control, New Zealand, 1988–2010. Emerg Infect Dis. 2012;18:226–33. PubMed http://dx.doi.org/10.3201/eid1802.111126

5. Siegler R, Oakes R. Hemolytic uremic syndrome; pathogenesis, treatment, and outcome. Curr Opin Pediatr. 2005;17:200–4. PubMed http://dx.doi.org/10.1097/01.mop.0000152997.66070.e9

6. Elliott EJ, Robins-Browne RM. Hemolytic uremic syndrome. Curr Probl Pediatr Adolesc Health Care. 2005;35:310–30. PubMed http://dx.doi.org/10.1016/j.cppeds.2005.06.002

7. Walker CL, Applegate JA, Black RE. Haemolytic-uraemic syndrome as a sequela of diarrhoeal disease. J Health Popul Nutr. 2012;30:257–61. PubMed http://dx.doi.org/10.3329/jhpn.v30i3.12288

8. Alvarado AS, Brodsky SV, Nadasdy T, Singh N. Hemolytic uremic syndrome associated with *Clostridium difficile* infection. Clin Nephrol. 2014;81:302–6. PubMed

9. Albaqali A, Ghuloom A, Al Arrayed A, Al Ajami A, Shome DK, Jamsheer A, et al. Hemolytic uremic syndrome in association with typhoid fever. Am J Kidney Dis. 2003;41:709–13. PubMed http://dx.doi.org/10.1053/ajkd.2003.50135

10. Baker NM, Mills AE, Rachman I, Thomas JE. Haemolytic-uraemic syndrome in typhoid fever. BMJ. 1974;2:84–7. PubMed http://dx.doi.org/10.1136/bmj.2.5910.84

11. McPherson M, Kirk MD, Raupach J, Coombs B, Butler JR. Economic costs of Shiga toxin–producing *Escherichia coli* infection in Australia. Foodborne Pathog Dis. 2011;8:55–62. PubMed http://dx.doi.org/10.1089/fpd.2010.0608

12. Mead PS, Griffin PM. *Escherichia coli* O157:H7. Lancet. 1998;352:1207–12. PubMed http://dx.doi.org/10.1016/S0140-6736(98)01267-7

13. Beutin L, Zimmerman S, Gleier K. Human infections with Shiga toxin–producing *Escherichia coli* other than serogroup O157 in Germany. Emerg Infect Dis. 1998;4:635–9. PubMed http://dx.doi.org/10.3201/eid0404.980415

14. McPherson M, Lalor K, Combs B, Raupach J, Stafford R, Kirk MD. Serogroup-specific risk factors for Shiga toxin–producing *Escherichia coli* infection in Australia. Clin Infect Dis. 2009;49:249–56. PubMed http://dx.doi.org/10.1086/599370

15. Vally H, Hall G, Dyda A, Raupach J, Knope K, Combs B, et al. Epidemiology of Shiga toxin producing *Escherichia coli* in Australia, 2000–2010. BMC Public Health. 2012;12:63–71. PubMed http://dx.doi.org/10.1186/1471-2458-12-63

16. Elliott EJ, Robins-Browne RM, O'Loughlin EV, Bennett-Wood V, Bourke J, Henning P, et al. Nationwide study of haemolytic uraemic syndrome: clinical, microbiological, and epidemiological features. Arch Dis Child. 2001;85:125–31. PubMed http://dx.doi.org/10.1136/adc.85.2.125

17. López EL, Contrini MM, Glastein E, Ayala SG, Santoro R, Ezcurra G, et al. An epidemiologic surveillance of Shiga-like toxin producing *Escherichia coli* infection in Argentinean children: risk factors and serum Shiga-like toxin 2 values. Pediatr Infect Dis J. 2012;31:20–4. PubMed http://dx.doi.org/10.1097/INF.0b013e31822ea6cf

18. Van Duynhoven YT, De Jager CM, Heuvelink AE, Van Der Zwaluw WK, Maas HM, Van Pelt W, et al. Enhanced laboratory-based surveillance of Shiga-toxin–producing *Escherichia coli* O157 in the Netherlands. Eur J Clin Microbiol Infect Dis. 2002;21:513–22. PubMed http://dx.doi.org/10.1007/s10096-002-0756-7

19. Käppeli U, Hächler H, Giezendanner N, Cheasty T, Stephan R. Shiga toxin–producing *Escherichia coli* O157 associated with human infections in Switzerland, 2000–2009. Epidemiol Infect. 2011;139:1097–104. PubMed

20. Bradley KK, Williams JM, Burnsed LJ, Lytle MB, McDermott MD, Mody RK, et al. Epidemiology of a large restaurant-associated outbreak of Shiga toxin–producing *Escherichia coli* O111:NM. Epidemiol Infect. 2012;140:1644–54. PubMed http://dx.doi.org/10.1017/S0950268811002329

21. Neil KP, Biggerstaff G, MacDonald JK, Trees E, Medus C, Musser KA, et al. a novel vehicle for transmission of *Escherichia coli* O157:H7 to humans: multistate outbreak of *E. coli* O157:H7 infections associated with consumption of ready-to-bake commercial prepackaged cookie dough—United States, 2009. Clin Infect Dis. 2012;54:511–8. PubMed http://dx.doi.org/10.1093/cid/cir831

22. Frank C, Werber D, Cramer JP, Askar M, Faber M, an der Heiden M, et al. Epidemic profile of Shiga-toxin–producing *Escherichia coli* O104:H4 outbreak in Germany. N Engl J Med. 2011;365:1771–80. PubMed http://dx.doi.org/10.1056/NEJMoa1106483

23. Jay MT, Garrett V, Mohle-Boetani JC, Barros M, Farrar JA, Rios R, et al. A multistate outbreak of *Escherichia coli* O157:H7 infection linked to consumption of beef tacos at a fast-food restaurant chain. Clin Infect Dis. 2004;39:1–7. PubMed http://dx.doi.org/10.1086/421088

24. Sigmundsdottir G, Atladottir A, Hardadottir H, Gudmundsdottir E, Geirsdottir M, Briem H. STEC O157 outbreak in Iceland, September–October 2007. Euro Surveill. 2007;12:E0711012. PubMed

25. Rangel JM, Sparling PH, Crowe C, Griffin PM, Swerdlow DL. Epidemiology of *Escherichia coli* O157:H7 outbreaks, United States, 1982–2002. Emerg Infect Dis. 2005;11:603–9. PubMed http://dx.doi.org/10.3201/eid1104.040739

26. Schwille-Kiuntke J, Frick JS, Zanger P, Enck P. Post-infectious irritable bowel syndrome—a review of the literature. Z Gastroenterol. 2011;49:997–1003. PubMed http://dx.doi.org/10.1055/s-0031-1281581

27. Haagsma JA, Siersema PD, De Wit NJ, Havelaar AH. Disease burden of post-infectious irritable bowel syndrome in the Netherlands. Epidemiol Infect. 2010;138:1650–6. PubMed http://dx.doi.org/10.1017/S0950268810000531

28. Thabane M, Kottachchi DT, Marshall JK. Systematic review and meta-analysis: the incidence and prognosis of post-infectious irritable bowel syndrome. Aliment Pharmacol Ther. 2007;26:535–44. PubMed http://dx.doi.org/10.1111/j.1365-2036.2007.03399.x

29. Halvorson HA, Schlett CD, Riddle MS. Postinfectious irritable bowel syndrome—a meta-analysis. Am J Gastroenterol. 2006;101:1894–9. PubMed http://dx.doi.org/10.1111/j.1572-0241.2006.00654.x

30. Smith JL, Bayles D. Postinfectious irritable bowel syndrome: a long-term consequence of bacterial gastroenteritis. J Food Prot. 2007;70:1762–9. PubMed

31. Neal KR, Hebden J, Spiller R. Prevalence of gastrointestinal symptoms six months after bacterial gastroenteritis and risk factors for development of the irritable bowel syndrome: postal survey of patients. BMJ. 1997;314:779–82. PubMed http://dx.doi.org/10.1136/bmj.314.7083.779

32. Parry SD, Stansfield R, Jelley D, Gregory W, Phillips E, Barton JR, et al. Does bacterial gastroenteritis predispose people to functional gastrointestinal disorders? A prospective, community-based, case–control study. Am J Gastroenterol. 2003;98:1970–5. PubMed

33. Mearin F, Perez-Oliveras M, Perello A, Vinyet J, Ibanez A, Coderch J, et al. Dyspepsia and irritable bowel syndrome after a *Salmonella* gastroenteritis outbreak: one-year follow-up cohort study. Gastroenterology. 2005;129:98–104. PubMed http://dx.doi.org/10.1053/j.gastro.2005.04.012

34. Ji S, Park H, Lee D, Song YK, Choi JP, Lee SI. Post-infectious irritable bowel syndrome in patients with *Shigella* infection. J Gastroenterol Hepatol. 2005;20:381–6. PubMed http://dx.doi.org/10.1111/j.1440-1746.2005.03574.x

35. Marshall JK. Post-infectious irritable bowel syndrome following water contamination. Kidney Int Suppl. 2009;Feb(112):S42–3. PubMed http://dx.doi.org/10.1038/ki.2008.618

36. Thabane M, Simunovic M, Akhtar-Danesh N, Garg AX, Clark WF, Collins SM, et al. An outbreak of acute bacterial gastroenteritis is associated with an increased incidence of irritable bowel syndrome in children. Am J Gastroenterol. 2010;105:933–9. PubMed http://dx.doi.org/10.1038/ajg.2010.74

37. Wensaas KA, Langeland N, Hanevik K, Morch K, Eide GE, Rortveit G. Irritable bowel syndrome and chronic fatigue 3 years after acute giardiasis: historic cohort study. Gut. 2012;61:214–9. PubMed http://dx.doi.org/10.1136/gutjnl-2011-300220

38. Penrose AS, Wells EV, Aiello AE. Infectious causation of chronic disease: examining the relationship between *Giardia lamblia* infection and irritable bowel syndrome. World J Gastroenterol. 2007;13:4574–8. PubMed

39. D’Anchino M, Orlando D, De Feudis L. *Giardia lamblia* infections become clinically evident by eliciting symptoms of irritable bowel syndrome. J Infect. 2002;45:169–72. PubMed http://dx.doi.org/10.1016/S0163-4453(02)91038-8

40. Marshall JK, Thabane M, Borgaonkar MR, James C. Postinfectious irritable bowel syndrome after a food-borne outbreak of acute gastroenteritis attributed to a viral pathogen. Clin Gastroenterol Hepatol. 2007;5:457–60. PubMed http://dx.doi.org/10.1016/j.cgh.2006.11.025

41. Koh SJ, Lee DH, Lee SH, Park YS, Hwang JH, Kim JW, et al. Incidence and risk factors of irritable bowel syndrome in community subjects with culture-proven bacterial gastroenteritis. Korean J Gastroenterol. 2012;60:13–8. PubMed http://dx.doi.org/10.4166/kjg.2012.60.1.13

42. Hannu T. Reactive arthritis. Best Pract Res Clin Rheumatol. 2011;25:347–57. PubMed http://dx.doi.org/10.1016/j.berh.2011.01.018

43. Townes JM. Reactive arthritis after enteric infections in the United States: the problem of definition. Clin Infect Dis. 2010;50:247–54. PubMed http://dx.doi.org/10.1086/649540

44. Girschick HJ, Guilherme L, Inman RD, Latsch K, Rihl M, Sherer Y, et al. Bacterial triggers and autoimmunie rheumatic diseases. Clin Exp Rheumatol. 2008;26(Suppl 48):S12–7. PubMed

45. Schönberg-Norio D, Mattila L, Lauhio A, Katila ML, Kaukoranta SS, Koskela M, et al. Patient-reported complications associated with *Campylobacter jejuni* infection. Epidemiol Infect. 2010;138:1004–11. PubMed http://dx.doi.org/10.1017/S0950268809991099

46. Doorduyn Y, Van Pelt W, Siezen CL, Van Der Horst F, Van Duynhoven YT, Hoebee B, et al. Novel insight in the association between salmonellosis or campylobacteriosis and chronic illness, and the role of host genetics in susceptibility to these diseases. Epidemiol Infect. 2008;136:1225–34. PubMed http://dx.doi.org/10.1017/S095026880700996X

47. Townes JM, Deodhar AA, Laine ES, Smith K, Krug HE, Barkhuizen A, et al. Reactive arthritis following culture-confirmed infections with bacterial enteric pathogens in Minnesota and Oregon: a population-based study. Ann Rheum Dis. 2008;67:1689–96. PubMed http://dx.doi.org/10.1136/ard.2007.083451

48. Schiellerup P, Krogfelt KA, Locht H. A comparison of self-reported joint symptoms following infection with different enteric pathogens: effect of HLA-B27. J Rheumatol. 2008;35:480–7. PubMed

49. Pope JE, Krizova A, Garg AX, Thiessen-Philbrook H, Ouimet JM. *Campylobacter* reactive arthritis: a systematic review. Semin Arthritis Rheum. 2007;37:48–55. PubMed http://dx.doi.org/10.1016/j.semarthrit.2006.12.006

50. Rees JR, Pannier MA, McNees A, Shallow S, Angulo FJ, Vugia DJ. Persistent diarrhea, arthritis, and other complications of enteric infections: a pilot survey based on California FoodNet

surveillance, 1998–1999. Clin Infect Dis. 2004;38(Suppl 3):S311–7. PubMed http://dx.doi.org/10.1086/381601

51. Hannu T, Mattila L, Rautelin H, Pelkonen P, Lahdenne P, Siitonen A, et al. *Campylobacter*-triggered reactive arthritis: a population-based study. Rheumatology (Oxford). 2002;41:312–8. PubMed http://dx.doi.org/10.1093/rheumatology/41.3.312

52. Locht H, Krogfelt KA. Comparison of rheumatological and gastrointestinal symptoms after infection with *Campylobacter jejuni*/*coli* and enterotoxigenic *Escherichia coli*. Ann Rheum Dis. 2002;61:448–52. PubMed http://dx.doi.org/10.1136/ard.61.5.448

53. Arnedo-Pena A, Beltran-Fabregat J, Vila-Pastor B, Tirado-Balaguer MD, Herrero-Carot C, Bellido-Blasco JB, et al. Reactive arthritis and other musculoskeletal sequelae following an outbreak of *Salmonella* Hadar in Castellon, Spain. J Rheumatol. 2010;37:1735–42. PubMed http://dx.doi.org/10.3899/jrheum.091250

54. Lee AT, Hall RG, Pile KD. Reactive joint symptoms following an outbreak of *Salmonella* Typhimurium phage type 135a. J Rheumatol. 2005;32:524–7. PubMed

55. Buxton JA, Fyfe M, Berger S, Cox MB, Northcott KA. Reactive arthritis and other sequelae following sporadic *Salmonella* Typhimurium infection in British Columbia, Canada: a case control study. J Rheumatol. 2002;29:2154–8. PubMed

56. Hannu T, Mattila L, Siitonen A, Leirisalo-Repo M. Reactive arthritis following an outbreak of *Salmonella* Typhimurium phage type 193 infection. Ann Rheum Dis. 2002;61:264–6. PubMed http://dx.doi.org/10.1136/ard.61.3.264

57. Rudwaleit M, Richter S, Braun J, Sieper J. Low incidence of reactive arthritis in children following a *Salmonella* outbreak. Ann Rheum Dis. 2001;60:1055–7. PubMed http://dx.doi.org/10.1136/ard.60.11.1055

58. Urfer E, Rossier P, Mean F, Krending MJ, Burnens A, Bille J, et al. Outbreak of *Salmonella* Braenderup gastroenteritis due to contaminated meat pies: clinical and molecular epidemiology. Clin Microbiol Infect. 2000;6:536–42. PubMed http://dx.doi.org/10.1046/j.1469-0691.2000.00148.x

59. Huovinen E, Sihvonen LM, Virtanen MJ, Haukka K, Siitonen A, Kuusi M. Symptoms and sources of *Yersinia enterocolitica*-infection: a case–control study. BMC Infect Dis. 2010;10:122. PubMed http://dx.doi.org/10.1186/1471-2334-10-122

60. Hannu T, Mattila L, Nuorti JP, Ruutu P, Mikkola J, Siitonen A, et al. Reactive arthritis after an outbreak of *Yersinia pseudotuberculosis* serotype O:3 infection. Ann Rheum Dis. 2003;62:866–9. PubMed http://dx.doi.org/10.1136/ard.62.9.866
