# Supplementary material for: Sequelae of Foodborne Illness Caused by 5 Pathogens, Australia, Circa 2010
Source: Emerg Infect Dis. 2014 Nov;20(11):1865–71. doi: 10.3201/eid2011.131316 (PMC4214289; doi:10.3201/eid2011.131316)
Supplement: Technical Appendix 2 — Methods to estimate sequelae incidence. [file 13-1316-Techapp-s2.pdf]

Article DOI: http://dx.doi.org/10.3201/eid2011.131316

Sequelae of Foodborne Illness Caused by 5 Pathogens, Australia, Circa 2010

Technical Appendix 2

Methods to Estimate Sequelae Incidence

For all 4 sequelae illnesses, we used data from notifiable surveillance (either national or state notifications) to estimate incidence of acute gastroenteritis due to relevant pathogens and then adjusted this using a sequelae multiplier, which is the proportion of bacterial infections that lead to sequelae illnesses (online Technical Appendix 1, http://wwwnc.cdc.gov/EID/article/20/11/13-1316-Techapp1.pdf). This approach is displayed in the Technical Appendix 2 Figure, where the left-hand column describes each input or output distribution, the central column illustrates the distribution, and the right-hand column describes the type and source of data underlying each input distribution. The final estimate is produced from a statistical model that incorporates uncertainty in case numbers in multipliers using probability distributions. That is, at each stage of the calculation, the estimate is represented by a probability distribution, and our final estimates and credible intervals are computed from this distribution. Further details on the estimation of incidence of acute illness due to each of the causal pathogens can be found in Kirk et al. (*1*).

The sequelae multiplier was modelled by using the PERT (Project Evaluation and Review Techniques) distribution, which is widely used for expert elicitation and risk assessment studies. It is based on the beta distribution and allows the input of minimum, maximum, and modal values. The alternate PERT distribution can be specified by 3 percentile points, such as a median value and 95% credible intervals (CrIs). Alternate PERT was used for the hemolytic uremic syndrome and irritable bowel syndrome sequelae multiplier, as the multiplier used was from another study that used median and 95% CIs. Alternate PERT was also used for reactive arthritis sequelae multipliers to enable a median value to be input, except in the case of the *Shigella*-associated reactive arthritis, where an alternate PERT distribution would not fit the data, and a PERT distribution was used instead. PERT allows for asymmetric distributions and can be easily produced from many data sources.

Reference

1. Kirk M, Ford L, Glass K, Hall G. Foodborne illness, Australia, circa 2000–circa 2010. Emerg Infect Dis. 2014;20:zzz–zzz. http://dx.doi.org/10.3201/eid2011.131315

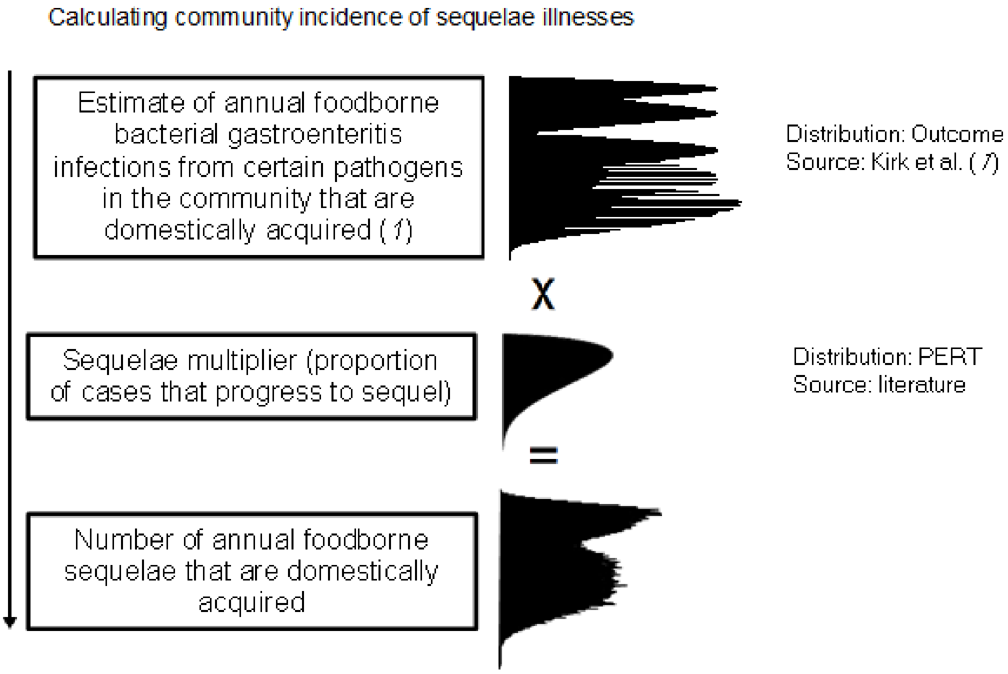

Technical Appendix 2 Figure. Flowchart for the approach used to calculate the estimated number of sequelae cases in the community, Australia, circa 2010. PERT, project evaluation and review technique.
