# Supplementary material for: Sequelae of Foodborne Illness Caused by 5 Pathogens, Australia, Circa 2010
Source: Emerg Infect Dis. 2014 Nov;20(11):1865–71. doi: 10.3201/eid2011.131316 (PMC4214289; doi:10.3201/eid2011.131316)
Supplement: Technical Appendix 3 — Comparison with estimates from 2000. [file 13-1316-Techapp-s3.pdf]

Article DOI: http://dx.doi.org/10.3201/eid2011.131316

Sequelae of Foodborne Illness Caused by 5 Pathogens, Australia, Circa 2010

Technical Appendix 3

Comparison with Estimates from 2000

Hall et al. estimated incidence, hospitalizations, and deaths for these 4 sequelae illnesses in Australia circa 2000 (*1*). Because methods and data sources have changed since the 2000 estimation effort, we recalculated incidence estimates for the sequelae in 2000 using our current methods and equivalent data from that earlier time period to validly compare rates over time. We used National Notifiable Disease Surveillance System data from 1996 to 2000 to recalculate the estimates for the incidence of all cases of gastroenteritis due to foodborne *Campylobacter* spp., nontyphoidal *Salmonella enterica* serotypes (hereafter referred to as nontyphoidal *Salmonella* spp.), *Shigella* spp., and *Yersinia enterocolitica* (*2*,*3*), and South Australian data from 1998–2000 (*3*) to recalculate the 2000 estimate for the incidence of gastroenteritis due to Shiga toxin–producing *Escherichia coli* (STEC). Further details on the method and recalculated circa 2000 estimates for *Campylobacter* spp., nontyphoidal *Salmonella* spp., and *Shigella* spp. can be found in the methods section and Table 3 of Kirk et al. (*4*). The estimates of foodborne illness from STEC and *Y. enterocolitica* for circa 2000 were calculated solely for this paper, using the same methods described in Kirk et al. (*4*) and the data described above.

Sequelae multipliers for the 2010 estimates were then applied to the recalculated 2000 estimates of incidence of acute gastroenteritis. The Technical Appendix 3 Table presents a comparison of the recalculated incidence estimates of sequelae of Guillain-Barré syndrome, hemolytic uremic syndrome, irritable bowel syndrome, and reactive arthritis for 2000 and 2010. Changes in sequelae illness from 2000 to 2010 reflect changes in the incidence of the preceding bacterial pathogen because the rate of sequelae after foodborne gastroenteritis, otherwise referred to as the sequelae multiplier, is assumed to be constant over this time period.

Technical Appendix 3 Table. Comparison of incidence estimates and rates of 4 sequelae, Australia, circa 2000 and 2010*

	2000		2010		
Illness	Incidence (90% CrI)	Rate per million (90% CrI)	Incidence (90% CrI)	Rate per million (90% CrI)	Rate ratio (90% CrI)
GBS	50 (25–100)	2.8 (1–6)	70 (30–150)	3.1 (2–6)	1.13 (0.5–3.6)
HUS	55 (15–175)	3 (1–9)	70 (25–200)	3 (1–9)	1 (0.3–3.5)
IBS	14,800 (9,500–23,500)	850 (550–1,350)	19,500 (12,500–30,700)	915 (570–1,440)	1.07 (0.5–2.0)
ReA	12,500 (6,700–23,000)	730 (380–1,325)	16,200 (8,750–30,400)	765 (415–1,375)	1.06 (0.4–2.5)

*GBS, Guillain-Barré syndrome; HUS, hemolytic uremic syndrome; IBS, irritable bowel syndrome; ReA, reactive arthritis; CrI, credible interval.

References

1. Hall G, Kirk M. Foodborne illnesses in Australia: annual incidence circa 2000. Report no. 0642825769. Canberra (ACT): Commonwealth Department of Health and Ageing; 2005.

2. Government of Australia. National Notifiable Disease Surveillance System (NNDSS). Commonwealth of Australia; 2013 [cited 2013 April 5]. http://www9.health.gov.au/cda/source/cda-index.cfm

3. Hall G. OzFoodNet Working Group. How much gastroenteritis in Australia is due to food? Report no. NCEPH working paper no. 51. Sponsored by the Commonwealth Department of Health and Ageing. Canberra (Australia): National Centre for Epidemiology and Population Health. 2004.

4. Kirk M, Ford L, Glass K, Hall G. Foodborne illness, Australia, circa 2000–circa 2010. Emerg Infect Dis. 2014;20:zzz–zzz. http://dx.doi.org/10.3201/eid2011.131315
