# Supplementary material for: Sequelae of Foodborne Illness Caused by 5 Pathogens, Australia, Circa 2010
Source: Emerg Infect Dis. 2014 Nov;20(11):1865–71. doi: 10.3201/eid2011.131316 (PMC4214289; doi:10.3201/eid2011.131316)
Supplement: Technical Appendix 4 — Methods to estimate sequelae hospitalizations and deaths. [file 13-1316-Techapp-s4.pdf]

Article DOI: http://dx.doi.org/10.3201/eid2011.131316

# Sequelae of Foodborne Illness Caused by 5 Pathogens, Australia, Circa 2010

## Technical Appendix 4

## Methods to Estimate Sequelae Hospitalizations and Deaths

To estimate hospitalizations due to irritable bowel syndrome (IBS) and reactive arthritis (ReA), we used hospitalization data for 2006–2010 from all Australian states and territories, using International Classification of Disease, Tenth Revision, Australian Modification (ICD-10-AM) codes. All estimated incident foodborne *Campylobacter*-associated Guillain-Barré syndrome (GBS) and Shiga toxin–producing *Escherichia coli* (STEC)–associated hemolytic uremic syndrome (HUS) cases were considered hospitalized, so were not modeled. The estimate for hospitalizations due to GBS and HUS is the estimate for GBS and HUS incidence. To estimate deaths for all 4 sequelae illnesses, we used national deaths data for 2001–2010 from the Australian Bureau of Statistics, using ICD-10 codes (Technical Appendix 4 Table 1). The final estimate included 2 multipliers, which are discussed below.

Technical Appendix 4 Table 1. Mortality and hospitalization codes for each sequel, Australia, 2010*

| Sequelae | Mortality ICD-10 code and description | Hospitalization ICD-10-AM code and description |
|---|---|---|
| Guillain-Barré syndrome | G610: Guillain-Barré syndrome | – |
| Hemolytic uremic syndrome | D593: Hemolytic uremic syndrome | – |
| Irritable bowel syndrome | K58: Irritable bowel syndrome | K58.0: Irritable bowel syndrome with diarrhea<br>K58.9: Irritable bowel syndrome without diarrhea |
| Reactive arthritis | M021: Postdysenteric arthropathy<br>M028: Other reactive arthropathies | M02.1: Postdysenteric arthropathy<br>M02.3: Reiter's disease<br>M02.8: Other reactive arthropathies<br>M03.2: Other postinfectious arthropathies in diseases classified elsewhere |

*ICD-10-AM, International Classification of Diseases, Tenth Revision; AM, Australian Modification; –, all patients with incident cases are assumed to have been hospitalized so hospitalization data not used for this pathogen.

### Domestically Acquired Multiplier

This multiplier adjusts for the proportion of case-patients who acquired infection in Australia with values for each sequelae in Technical Appendix 4 Table 2. For GBS, we adopted the domestically acquired multiplier for *Campylobacter* spp. (*1*). Given the relatively small numbers of notified cases of HUS, we adopted the domestically acquired multiplier for STEC (*1*). The domestically acquired multiplier for IBS was calculated as a weighted average of the

domestically acquired multipliers for *Campylobacter* spp., nontyphoidal *Salmonella enterica* serotypes (hereafter referred to as nontyphoidal *Salmonella* spp.), and *Shigella* spp., weighted by the total number of IBS cases for each pathogen. Similarly, the domestically acquired multiplier for ReA was calculated as a weighted average of the domestically acquired multipliers for *Campylobacter* spp., nontyphoidal *Salmonella* spp., *Shigella* spp., and *Yersinia enterocolitica*, weighted by the total number of ReA cases for each pathogen.

Technical Appendix 4 Table 2. Domestically acquired multipliers*

| Sequelae | Domestically acquired multiplier |
|---|---|
| Guillain-Barré syndrome | 0.97 (range 0.91–0.99) |
| Hemolytic uremic syndrome | 0.99 (range 0.93–1.00) |
| Irritable bowel syndrome | 0.91 (90% CrI 0.88–0.94) |
| Reactive arthritis | 0.91 (90% CrI 0.86–0.95) |

*CrI, credible interval.

### Proportion Foodborne Multiplier

This multiplier adjusts for the proportion of illness that is acquired from food and was required only to estimate hospitalizations and deaths. Sequelae can arise from a source other than a bacterial pathogen, from a bacterial pathogen that was not foodborne, or from a foodborne pathogen. Only this latter category is considered a foodborne source. The proportion foodborne multiplier is the simulated product of the bacterial multiplier and the weighted foodborne multiplier and can be found in Technical Appendix 4 Table 3. The approach for calculating the proportion foodborne multiplier for each sequel is described as follows:

Technical Appendix 4 Table 3. Proportion foodborne multiplier*

| Sequelae | Foodborne multiplier |
|---|---|
| Guillain-Barré syndrome | 0.25 (90% CrI 0.1–0.43) |
| Hemolytic uremic syndrome | 0.33 (90% CrI 0.17–0.53) |
| Irritable bowel syndrome | 0.13 (90% CrI 0.08–0.20) |
| Reactive arthritis | 0.48 (90% CrI 0.36–0.62) |

*CrI, credible interval.

#### GBS

There have been several reviews, as well as many case–control and cross-sectional studies, that estimated the percentage of GBS cases attributable to *Campylobacter* spp. (Technical Appendix 4 Table 3). Poropatich et al. (*8*) performed a systematic review of 30 case–control studies and concluded that 31.0% of GBS cases might be attributable to a previous infection due to *Campylobacter* spp. (*8*). The other global systematic review of GBS incidence does not look at *Campylobacter* spp. specifically or perform a meta-analysis (*9*). Other (nonsystematic) reviews have found that 13%–72% (*10*) and 8%–50% (*11*) of GBS occurs as a sequel to campylobacteriosis. We assume that 31% (range 4.8%–72%) of cases of GBS arise

from *Campylobacter* spp. (*2*). Multiplied together with the *Campylobacter* spp. foodborne multiplier of 0.77 (90% CrI 0.62–0.89) (*1*) led to a foodborne multiplier for GBS of 0.25 (90% CrI 0.11–0.43).

Technical Appendix 4 Table 4. Proportion of Guillain-Barré syndrome attributable to *Campylobacter* spp.*

| Reference | Study years | Country | Study type | No. GBS cases | No. *Campylobacter* spp. cases based on | GBS cases attributable to campylobacteriosis |
|---|---|---|---|---|---|---|
| **Poropatich et al. (*8*)** | **1982–2010** | **Global** | **Systematic review** | **2,502** | **Stool samples or serology** | **31% (range 4.8%–71.7%)** |
| McGrogan et al. (*9*) | 1980–2008 | Global | Systematic review | – | – | 6%–26% |
| Islam et al. (*12*) | 2006–2007 | Bangladesh | Prospective case-control | 100 | Stool samples and serology | 57% |
| Sivadon-Tardy et al. (*13*) | 1999–2005 | France | Cross sectional | 237 | Stool samples and serology | 27% |
| Tam et al. (*14*) | 1991–2001 | UK | Nested case-control | 553 | Corrected community incidence estimate | 20% |
| Sivadon-Tardy et al. (*15*) | 1996–2001 | France | Cross sectional | 263 | Serology | 22% |
| Takahashi et al. (*16*) | 1990–2003 | Japan | Case-control | 1049 | Stool samples and serology | 11% |
| Tam et al. (*17*) | 2000–2001 | UK | Estimation | 1146 | Community incidence estimate | 13.7% |
| Hadden and Gregson (*10*) | – | Global | Review | – | Serology | 13%–72% |
| Nachamkin et al. (*11*) | – | USA | Review | – | Stool samples or serology | Best estimate 30%–40% (range 8%–50%) |

*Boldface indicates chosen proportion for foodborne multiplier calculation.

HUS

Technical Appendix 4 Table 5 presents the percentage of cases of HUS that arise from STEC estimated in 4 different papers, including a global systematic review. From this, we assumed that 61% (range 30%–85%) of HUS cases arise from STEC, modelled as a PERT distribution. Multiplied with the STEC foodborne multiplier of 0.56 (90% credible interval [CrI] 0.32–0.83) (*1*) led to a foodborne multiplier for HUS of 0.33 (90% CrI 0.18–0.54).

Technical Appendix 4 Table 5. Proportion of HUS attributable to STEC*

| Reference | Study years | Study type | Country | No. STEC isolations/no. HUS cases | STEC cases that develop into HUS |
|---|---|---|---|---|---|
| **Walker et al. (*18*)** | **1980–2011** | **Systematic review** | **Global** | **–** | **60.8% (range 30%–85.2%)** |
| Askar et al. (*19*) | 2011 | Surveillance | Germany | 273/470 | 58% |
| Elliot et al. (*20*) | 1994–1998 | Surveillance | Australia | 36/70 | 51% |
| Van de Kar (*21*) | 1989–1993 | Case control | The Netherlands | 88/113 | 77.8% |

*HUS, hemolytic uremic syndrome; STEC, Shiga toxin–producing *Escherichia coli*. Boldface indicates chosen proportion for foodborne multiplier calculation.

IBS

We estimated the proportion of IBS cases from *Campylobacter* spp., nontyphoidal *Salmonella* spp., or *Shigella* spp. based on the proportion of IBS considered to be postinfectious in the literature. In 1962, Chaudhary and Truelove (*22*) reported IBS occurring from infective dysentery, with 34 (26.2%) of 130 patients dating symptoms back to an attack of gastroenteritis.

More recently, review studies have estimated that 6%-17% (*23*) and 7%–33% of IBS is postinfectious (*24*). In the meta-analysis and estimation by Haagsma et al. (*25*), the authors considered that 17% of IBS is due to campylobacteriosis, salmonellosis, or shigellosis from the top end of the range of 6%-17% by Spiller and Garsed (*23*). We assumed 17% of IBS to be triggered by a gastrointestinal infection (*25*), with a range of 7%–33% from the review by Schwille-Kiuntke et al. (*24*). Because more than just *Campylobacter* spp., nontyphoidal *Salmonella* spp. and *Shigella* spp. can cause postinfectious IBS, this may be an overestimate.

A foodborne multiplier for the combined 3 pathogens of 73% (90% CrI 64%–82%) was calculated as a weighted average of the foodborne multipliers for each pathogen, weighted by the total number of IBS cases for each pathogen. Multiplied by the above PERT distribution of 17% (range 6%–33%), gave a foodborne multiplier for IBS of 13% (90% CrI 8%–20%).

Technical Appendix 4 Table 6. Proportion of IBS attributable to infectious gastroenteritis*

| Reference | Publication year | Study type | Country | No. postinfectious IBS cases/IBS cases | IBS that is postinfectious, % |
|---|---|---|---|---|---|
| Chaudhary and Truelove (*22*) | 1962 | Epidemiologic report | UK | 34/130 | 26.2 |
| Spiller and Garsed (*23*) | 2009 | Review | Global | – | 6–17 |
| **Haagsma et al. (*25*)** | **2010** | **Meta-analysis and estimation** | **The Netherlands** | **–** | **17** |
| **Schwille-Kiuntke et al. (*24*)** | **2013** | **Review** | **Global** | **–** | **7–33** |

*IBS, irritable bowel syndrome. Boldface indicates chosen proportion for foodborne multiplier calculation.

### ReA

In a review of ReA, Hannu et al. (*4*) compiled population-based studies on the annual incidence of ReA—both from enteric and urogenital infection. We used this compilation and calculated the proportion of ReA due to enteric infection by dividing the enteric incidence by the total incidence found in each study (Technical Appendix 4 Table 7). We used the midpoint and range of the proportions from these studies for the bacterial multiplier. We therefore assumed a median of 66.7% of ReA is due to an enteric infection, with a range of 50%–94.7%. If enteric infections preceding ReA are from other infections besides campylobacteriosis, salmonellosis, shigellosis, or yersiniosis, using this distribution to estimate ReA cases from these infections may cause an overestimation.

We adjusted for the proportion foodborne using a weighted average of the foodborne multipliers for *Campylobacter* spp., nontyphoidal *Salmonella* spp., *Shigella* spp., and *Y. enterocolitica,* weighted by the total number of ReA cases for each pathogen. This gave a foodborne multiplier of 72% (90% CrI 60%–82%). Multiplied by the above alternate PERT

distribution of median 66.7% (range 50%–94.7%), gave a foodborne multiplier for reactive arthritis of 48% (90% CrI 36%–61%).

Technical Appendix 3 Table 7. Proportion of ReA attributable to enteric infection*

| Reference | Country | Year | Incidence per 100,000 | | | No. ReA due to enteric infection/total no. enteric infections |
|---|---|---|---|---|---|---|
| | | | Enteric | Urogenital | Total | |
| Isomaki et al. (*26*) | Finland | 1978 | 14 | 13 | 27 | 14/27 (51.9%) |
| Kvien et al. (*27*) | Norway | 1994 | 5 | 5 | 10 | 5/10 (50%) |
| Savolainen et al. (*28*) | Finland | 2000 | 7 | 3 | 10 | 7/10 (70%) |
| Soderlin et al. (*29*) | Sweden | 2002 | 18 | 1 | 19 | 18/19 (94.7%) |
| Townes et al. (*30*) | USA | 2008 | 0.6–3.1 | NA | NA | NA |
| Hanova et al. (*31*) | Czech Republic | 2010 | 6 | 3 | ≈9 | 6/9 (66.7%) |

*Adapted from the table of annual incidence of reactive arthritis based on population studies in Hannu et al. (*4*). NA, not applicable.

## References


1. Kirk M, Ford L, Glass K, Hall G. Foodborne illness, Australia, circa 2000–circa 2010. Emerg Infect Dis. 2014;20:zzz–zzz. http://dx.doi.org/10.3201/eid2011.131315

2. Olden KW. Diagnosis of irritable bowel syndrome. Gastroenterology. 2002;122:1701–14. PubMed http://dx.doi.org/10.1053/gast.2002.33741

3. Townes JM. Reactive arthritis after enteric infections in the United States: the problem of definition. Clin Infect Dis. 2010;50:247–54. PubMed http://dx.doi.org/10.1086/649540

4. Hannu T. Reactive arthritis. Best Pract Res Clin Rheumatol. 2011;25:347–57. PubMed http://dx.doi.org/10.1016/j.berh.2011.01.018

5. Hall G, Kirk M, Becker N, Gregory J, Unicomb L, Millard G, et al. Estimating foodborne gastroenteritis, Australia. Emerg Infect Dis. 2005;11:1257–64. PubMed http://dx.doi.org/10.3201/eid1108.041367

6. Scallan E, Hoekstra RM, Angulo FJ, Tauxe RV, Widdowson MA, Roy SL, et al. Foodborne illness acquired in the United States—major pathogens. Emerg Infect Dis. 2011;17:7–15. PubMed http://dx.doi.org/10.3201/eid1701.P11101

7. Mead PS, Slutsker L, Dietz V, McCraig LF, Bresee JS, Shapiro C, et al. Food-related illness and death in the United States. Emerg Infect Dis. 1999;5:607–25. PubMed http://dx.doi.org/10.3201/eid0505.990502

8. Poropatich KO, Walker CL, Black RE. Quantifying the association between *Campylobacter* infection and Guillain-Barré syndrome: a systematic review. J Health Popul Nutr. 2010;28:545–52. PubMed http://dx.doi.org/10.3329/jhpn.v28i6.6602

9. McGrogan A, Madle GC, Seaman HE, de Vries CS. The epidemiology of Guillain-Barré syndrome worldwide. A systematic literature review. Neuroepidemiology. 2009;32:150–63. PubMed http://dx.doi.org/10.1159/000184748

10. Hadden RD, Gregson NA. Guillain–Barré syndrome and *Campylobacter jejuni* infection. Symp Ser Soc Appl Microbiol. 2001;(30):145S–54S. PubMed http://dx.doi.org/10.1046/j.1365-2672.2001.01363.x

11. Nachamkin I, Allos BM, Ho T. *Campylobacter* species and Guillain-Barré syndrome. Clin Microbiol Rev. 1998;11:555–67. PubMed

12. Islam Z, Jacobs BC, van Belkum A, Mohammad QD, Islam MB, Herbrink P, et al. Axonal variant of Guillain-Barré syndrome associated with *Campylobacter* infection in Bangladesh. Neurology. 2010;74:581–7. PubMed http://dx.doi.org/10.1212/WNL.0b013e3181cff735

13. Sivadon-Tardy V, Orlikowski D, Porcher R, Ronco E, Caudie C, Roussi J, et al. Guillain-Barré syndrome, greater Paris area. Emerg Infect Dis. 2006;12:990–3. PubMed

14. Tam CC, O'Brien SJ, Petersen I, Islam A, Hayward A, Rodrigues LC. Guillain-Barré syndrome and preceding infection with *Campylobacter*, influenza and Epstein-Barr virus in the general practice research database. PLoS ONE. 2007;2:e344. PubMed http://dx.doi.org/10.1371/journal.pone.0000344

15. Sivadon-Tardy V, Orlikowski D, Rozenberg F, Caudie C, Sharshar T, Lebon P, et al. Guillain-Barré syndrome, greater Paris area. Emerg Infect Dis. 2006;12:990–3. PubMed http://dx.doi.org/10.3201/eid1206.051369

16. Takahashi M, Koga M, Yokoyama K, Yuki N. Epidemiology of *Campylobacter jejuni* isolated from patients with Guillain-Barré and Fisher syndromes in Japan. J Clin Microbiol. 2005;43:335–9. PubMed http://dx.doi.org/10.1128/JCM.43.1.335-339.2005

17. Tam CC, O'Brien SJ, Adak GK, Meakins SM, Frost JA. *Campylobacter coli*—an important foodborne pathogen. J Infect. 2003;47:28–32. PubMed http://dx.doi.org/10.1016/S0163-4453(03)00042-2

18. Walker CL, Applegate JA, Black RE. Haemolytic-uraemic syndrome as a sequela of diarrhoeal disease. J Health Popul Nutr. 2012;30:257–61. PubMed http://dx.doi.org/10.3329/jhpn.v30i3.12288

19. Askar M, Faber MS, Frank C, Bernard H, Gilsdorf A, Fruth A, et al. Update on the ongoing outbreak of haemolytic uraemic syndrome due to Shiga toxin–producing *Escherichia coli* (STEC) serotype O104, Germany, May 2011. Euro Surveill. 2011;16: pii: 19883. PubMed

20. Elliott EJ, Robins-Browne RM, O''Loughlin EV, Bennett-Wood V, Bourke J, Henning P, et al. Nationwide study of haemolytic uraemic syndrome: clinical, microbiological, and epidemiological features. Arch Dis Child. 2001;85:125–31. PubMed http://dx.doi.org/10.1136/adc.85.2.125

21. van de Kar NC, Roelofs HG, Muytjens HL, Tolboom JJ, Roth B, Proesmans W, et al. Verocytotoxin-producing *Escherichia coli* infection in hemolytic uremic syndrome in part of western Europe. Eur J Pediatr. 1996;155:592–5. PubMed

22. Chaudhary NA, Truelove SC. The irritable colon syndrome: a study of the clinical features, predisposing causes, and prognosis in 130 cases. Q J Med. 1962;31:307–22. PubMed

23. Spiller R, Garsed K. Postinfectious irritable bowel syndrome. Gastroenterology. 2009;136:1979–88. PubMed http://dx.doi.org/10.1053/j.gastro.2009.02.074

24. Schwille-Kiuntke J, Frick JS, Zanger P, Enck P. Post-infectious irritable bowel syndrome—a review of the literature. Z Gastroenterol. 2011;49:997–1003. PubMed http://dx.doi.org/10.1055/s-0031-1281581

25. Haagsma JA, Siersema PD, De Wit NJ, Havelaar AH. Disease burden of post-infectious irritable bowel syndrome in the Netherlands. Epidemiol Infect. 2010;138:1650–6. PubMed http://dx.doi.org/10.1017/S0950268810000531

26. Isomäki H, Raunio J, von Essen R, Hämeenkorpi R. Incidence of inflammatory rheumatic diseases in Finland. Scand J Rheumatol. 1978;7:188–92. PubMed http://dx.doi.org/10.3109/03009747809095652

27. Kvien TK, Glennas A, Melby K, Granfors K, Andrup O, Karstensen B, et al. Reactive arthritis: incidence, triggering agents and clinical presentation. J Rheumatol. 1994;21:115–22. PubMed

28. Savolainen E, Kaipiainen-Seppanen O, Kroger L, Luosujarvi R. Total incidence and distribution of inflammatory joint diseases in a defined population: results from the Kuopio 2000 arthritis survey. J Rheumatol. 2003;30:2460–8. PubMed

29. Söderlin MK, Kautiainen H, Puolakkainen M, Hedman K, Söderlund-Venermo M, Skogh T, et al. Infections preceding early arthritis in southern Sweden: a prospective population-based study. J Rheumatol. 2003;30:459–64. PubMed

30. Townes JM, Deodahar AA, Laine ES, Smith K, Krug HE, Barkhuizen A, et al. Reactive arthritis following culture-confirmed infections with bacterial enteric pathogens in Minnesota and Oregon: a population-based study. Ann Rheum Dis. 2008;67:1689–96. PubMed http://dx.doi.org/10.1136/ard.2007.083451

31. Hanova P, Pavelka K, Holcatova I, Pikhart H. Incidence and prevalence of psoriatic arthritis, ankylosing spondylitis, and reactive arthritis in the first descriptive population-based study in the Czech Republic. Scand J Rheumatol. 2010;39:310–7. PubMed http://dx.doi.org/10.3109/03009740903544212f
