# Supplementary material for: Sequelae of Foodborne Illness Caused by 5 Pathogens, Australia, Circa 2010
Source: Emerg Infect Dis. 2014 Nov;20(11):1865–71. doi: 10.3201/eid2011.131316 (PMC4214289; doi:10.3201/eid2011.131316)
Supplement: Technical Appendix 5 — Model inputs for 4 sequelae illnesses due to contaminated food. [file 13-1316-Techapp-s5.pdf]

Article DOI: http://dx.doi.org/10.3201/eid2011.131316

Sequelae of Foodborne Illness Caused by 5 Pathogens, Australia, Circa 2010

Technical Appendix 5

Model Inputs for 4 Sequelae Illnesses Due to Contaminated Food

Incidence

Technical Appendix 5 Table 1. Guillain-Barré Syndrome

Model input, source, and comments	Distribution	Data for model input
Antecedent bacterial gastroenteritis cases: estimated number of foodborne *Campylobacter* spp. cases (*1*)	Outcome	5%, median, 95% values:<br>108500, 179000, 290000 (circa 2010)<br>82500, 139000, 227000 (circa 2000)
Sequelae multiplier: this proportion was a midpoint between estimates from the literature reported in Tam et al. (*2*), McCarthy and Gieseke (*3*), and Allos et al. (*4*)	PERT	Minimum, modal, maximum values:<br>0.000192, 0.000304, 0.000945
Total foodborne illness: foodborne *Campylobacter* spp. cases × Sequelae multiplier	Outcome	5%, median, 95% values:<br>30, 75, 150 (circa 2010)<br>25, 50, 100 (circa 2000)
Rate of foodborne illness from *Campylobacter* spp. per million population	Outcome	5%, median, 95% values:<br>2, 3.1, 6 (circa 2010)<br>1, 2.8, 6 (circa 2000)

Technical Appendix 5 Table 2. Hemolytic uremic syndrome

Model input, source, and comments	Distribution	Data for model input
Antecedent bacterial gastroenteritis cases: estimated number of foodborne STEC cases (*1*)	Outcome	5%, median, 95% values:<br>950, 2350, 5850 (circa 2010)<br>550, 1900, 5000 (circa 2000)
Sequelae multiplier: this proportion is from Vally et al. (*5*)	Alternate PERT	2.5%, Median, 97.5% values:<br>0.017, 0.03, 0.051
Total foodborne illness: foodborne STEC cases × Sequelae multiplier	Outcome	5%, median, 95% values:<br>25, 70, 200 (circa 2010)<br>15, 55, 175 (circa 2000)
Rate of foodborne illness from STEC per million	Outcome	5%, median, 95% values:<br>1, 3.3, 9 (circa 2010)<br>1, 3.0, 9 (circa 2000)

*STEC, Shiga toxin–producing *Escherichia coli*.

Technical Appendix 5 Table 3. Irritable bowel syndrome

Model input, source, and comments	Distribution	Data for model input
Antecedent bacterial gastroenteritis cases:		
Estimated number of foodborne *Campylobacter* spp. cases (*1*)	Outcome	5%, median, 95% values:<br>108500, 179000, 290000 (circa 2010)<br>82500, 139000, 227000 (circa 2000)
Estimated number of foodborne nontyphoidal *Salmonella* spp. cases (*1*)	Outcome	5%, median, 95% values:<br>21200, 39600, 73400 (circa 2010)<br>15000, 28000, 50000 (circa 2000)
Estimated number of foodborne *Shigella* spp. cases (*1*)	Outcome	5%, median, 95% values:<br>150, 350, 850 (circa 2010)<br>175, 515, 1300 (circa 2000)
Sequelae multiplier: This proportion was from Haagsmsa et al. (*6*)	Alternate PERT	2.5%, Median, 97.5% values:<br>0.072, 0.088, 0.104
Total foodborne illness<br>Foodborne *Campylobacter* spp. cases × Sequelae multiplier +	Outcome	5%, median, 95% values:<br>12500, 19500, 30700 (circa 2010)

Model input, source, and comments	Distribution	Data for model input
Foodborne nontyphoidal *Salmonella* spp. cases × Sequelae multiplier + Foodborne *Shigella* spp. cases × Sequelae multiplier		9500, 14800, 23500 (circa 2000)
Rate of foodborne illness per million	Outcome	5%, median, 95% values: 570, 915, 1440 (circa 2010) 550, 850, 1350 (circa 2000)

Technical Appendix 5 Table 4. Reactive arthritis

Model input, source, and comments	Distribution	Data for model input
Antecedent bacterial gastroenteritis cases:		
Estimated number of foodborne *Campylobacter* spp. cases (*1*)	Outcome	5%, median, 95% values: 108500, 179000, 290000 (circa 2010) 82500, 139000, 227000 (circa 2000)
Estimated number of foodborne nontyphoidal *Salmonella* spp. cases (*1*)	Outcome	5%, median, 95% values: 21200, 39600, 73400 (circa 2010) 15000, 28000, 50000 (circa 2000)
Estimated number of foodborne *Shigella* spp. cases (*1*)	Outcome	5%, median, 95% values: 150, 350, 850 (circa 2010) 175, 515, 1300 (circa 2000)
Estimated number of foodborne *Yersinia enterocolitica* cases (*1*)	Outcome	5%, median, 95% values: 650, 1150, 1950 (circa 2010) 300, 800, 1650 (circa 2000)
Sequelae multipliers: The proportion for each of the 4 pathogens was calculated from the literature. See Technical Appendix 1 for further explanation.		
Campylobacter spp. sequelae multiplier	Alternate PERT	Minimum, median, maximum values: 0.028, 0.07, 0.16
Nontyphoidal *Salmonella* spp. sequelae multiplier	Alternate PERT	Minimum, median, maximum values: 0, 0.085, 0.26
Shigella spp. sequelae multiplier	PERT	Minimum, modal, maximum values: 0.012, 0.097, 0.098
Yersinia enterocolitica sequelae multiplier	Alternate PERT	Minimum, median, maximum values: 0, 0.12, 0.231
Total foodborne illness: Foodborne *Campylobacter* spp. cases × Sequelae multiplier + Foodborne nontyphoidal *Salmonella* spp. cases × Sequelae multiplier + Foodborne *Shigella* spp. cases × Sequelae multiplier + Foodborne *Y. enterocolitica* cases × Sequelae multiplier	Outcome	5%, median, 95% values: 8750, 16200, 30400 (circa 2010) 6700, 12500, 23000 (circa 2000)
Rate of foodborne illness from *Campylobacter* spp., nontyphoidal *Salmonella* spp., *Shigella* spp. and *Y. enterocolitica* per million	Outcome	5%, median, 95% values: 415, 765, 1375 (circa 2010) 380, 730, 1325 (circa 2000)

Hospitalizations and Deaths

Technical Appendix 5 Table 5. Guillain-Barré syndrome

Model input, source, and comments	Distribution	Data for model input
Average number of deaths per year: Australian Bureau of Statistics death data	Empirical	2001–2010: 24.5
Population adjustment: Australian resident population June quarter, http://www.abs.gov.au/AUSSTATS/abs@.nsf/DetailsPage/3101.0Dec%202011?OpenDocument [cited 2012 Aug 16]	Empirical	By year (2001–2010): 19413240, 19651438, 19895435, 20127363, 20394791, 20697880, 21015936, 21384427, 21778845, 22065317
Domestically acquired multiplier: *Campylobacter* spp. domestic acquired multiplier	PERT	Minimum, modal, maximum values: 0.91, 0.97, 0.99
Foodborne multiplier: derived from:	Outcome	5%, median, 95% values: 0.1, 0.25, 0.43
Bacterial multiplier—the proportion of Guillain-Barré syndrome that is attributable to *Campylobacter* spp. from Poropatich et al. (*7*) ×	PERT	Minimum, modal, maximum values: 0.048, 0.31, 0.717
Campylobacter spp. foodborne proportion (*1*)	Alternate PERT	5%, median, 95% values: 0.62, 0.77, 0.89
Total foodborne deaths: circa 2010	Outcome	5%, median, 95% values: 2, 6, 10
Rate of foodborne deaths per million: circa 2010	Outcome	5%, median, 95% values: 0.1, 0.3, 0.5

Technical Appendix 5 Table 6. Hemolytic uremic syndrome*

Model input, source, and comments	Distribution	Data for model input

Model input, source, and comments	Distribution	Data for model input
Average number of deaths per year: Australian Bureau of Statistics death data	Empirical	2001–2010: 4.2
Population adjustment: Australian resident population June quarter, http://www.abs.gov.au/AUSSTATS/abs@.nsf/DetailsPage/3101.0Dec%202011?OpenDocument [cited 2012 Aug 16]	Empirical	By year (2001–2010):<br>19413240, 19651438, 19895435, 20127363, 20394791, 20697880, 21015936, 21384427, 21778845, 22065317
Domestically acquired multiplier: STEC domestically acquired multiplier	PERT	Minimum, modal, maximum values: 0.93, 0.9[illegible] 1
Foodborne multiplier:<br>derived from:<br>Bacterial multiplier—the proportion of HUS that is attributable to STEC from Walker et al. (*8*) × STEC foodborne proportion (*1*)	Outcome<br>PERT<br><br>Alternate PERT	5%, median, 95% values: 0.17, 0.33, 0.53<br>Minimum, modal, maximum values: 0.3, 0.608, 0.852<br>5%, median, 95% values: 0.32, 0.56, 0.83
Total foodborne deaths: circa 2010	Outcome	5%, median, 95% values: 1, 2, 3
Rate of foodborne deaths per million: circa 2010	Outcome	5%, median, 95% values: 0.03, 0.1, 0.12

*HUS, hemolytic uremic syndrome; STEC, Shiga toxin–producing *Escherichia coli*.

Technical Appendix 5 Table 7. Irritable bowel syndrome

Model input, source, and comments	Distribution	Data for model input
Yearly observed hospitalizations: state and territory hospitalization data<br>Average number of deaths per year: Australian Bureau of Statistics death data	Empirical<br><br>Empirical	By year (2006–2010): 7851, 7933, 7753, 8128, 7762<br>2001–2010: 13.1
Population adjustment: Australian resident population June quarter, http://www.abs.gov.au/AUSSTATS/abs@.nsf/DetailsPage/3101.0Dec%202011?OpenDocument [cited 2012 Aug 16]	Empirical	By year (2001–2010): 19413240, 19651438, 19895435, 20127363, 20394791, 20697880, 21015936, 21384427, 21778845, 22065317
Domestically acquired multiplier: a weighted multiplier from *Campylobacter* spp., nontyphoidal *Salmonella* spp., and *Shigella* spp. domestic multipliers	Alternate PERT	5%, median, 95% values: 0.88, 0.91, 0.94
Foodborne multiplier:<br>derived from:<br>Bacterial multiplier—proportion of IBS that is post-infectious extracted from the literature (*6,9*) × weighted *Campylobacter* spp., nontyphoidal *Salmonella* spp., and *Shigella* spp. foodborne proportion (*1*)	Outcome<br><br>Alternate PERT<br>Alternate PERT	5%, median, 95% values: 0.08, 0.13, 0.33<br><br>5%, median, 95% values: 0.06, 0.17, 0.33<br>5%, median, 95% values: 0.64, 0.73, 0.82
Total foodborne hospitalizations: circa 2010<br>Total foodborne deaths: circa 2010	Outcome<br>Outcome	5%, median, 95% values: 550, 915, 1400<br>5%, median, 95% values: 1, 2, 2
Rate of foodborne hospitalizations per million: circa 2010<br>Rate of foodborne deaths per million: circa 2010	Outcome<br>Outcome	5%, median, 95% values: 25, 43, 70<br>5%, median, 95% values: 0.05, 0.1, 0.11

Technical Appendix 5 Table 8. Reactive arthritis

Model input, source, and comments	Distribution	Data for model input
Yearly observed hospitalizations: State and Territory hospitalization data<br>Average number of deaths per year: Australian Bureau of Statistics death data	Empirical<br><br>Empirical	By year (2006–2010): 63, 50, 50, 70, 70<br><br>2001–2010: 0
Population adjustment: Australian resident population June quarter, http://www.abs.gov.au/AUSSTATS/abs@.nsf/DetailsPage/3101.0Dec%202011?OpenDocument [cited 2012 Aug 16]	Empirical	By year (2001–2010): 19413240, 19651438, 19895435, 20127363, 20394791, 20697880, 21015936, 21384427, 21778845, 22065317
Domestically acquired multiplier:<br>Weighted multiplier of *Campylobacter* spp., nontyphoidal *Salmonella* spp., *Shigella* spp., and *Y. enterocolitica* domestic multipliers	Alternate PERT	5%, median, 95% values:<br>0.86, 0.91, 0.95
Foodborne multiplier:<br>derived from:<br>Bacterial multiplier—proportion of ReA that is post-infectious extracted from the literature (*10*) × weighted *Campylobacter* spp., nontyphoidal *Salmonella* spp., and *Shigella* spp. foodborne proportion (*1*).	Alternate PERT<br><br>Alternate PERT<br>Alternate PERT	5%, median, 95% values:0.36, 0.48, 0.61<br><br>Minimum, median, maximum values: 0.5, 0.66, 0.947<br>5%, median, 95% values: 0.60, 0.72, 0.82
Total foodborne hospitalizations: circa 2010<br>Total foodborne deaths: circa 2010	Outcome<br>Outcome	5%, median, 95% values: 20, 25, 40<br>5%, median, 95% values: 0, 0, 0
Rate of foodborne hospitalizations per million: circa 2010<br>Rate of foodborne deaths per million: circa 2010	Outcome<br>Outcome	5%, median, 95% values: 1, 1, 2<br>5%, median, 95% values: 0, 0, 0

References

1. Kirk M, Ford L, Glass K, Hall G. Foodborne illness, Australia, circa 2000–circa 2010. Emerg Infect Dis. 2014;20:zzz–zzz. http://dx.doi.org/10.3201/eid2011.131315

2. McCarthy N, Giesecke J. Incidence of Guillain-Barré syndrome following infection with *Campylobacter jejuni*. Am J Epidemiol. 2001;153:610–4. PubMed http://dx.doi.org/10.1093/aje/153.6.610

3. Tam CC, Rodrigues LC, Petersen I, Islam A, Hayward A, O'Brien SJ. Incidence of Guillain-Barré syndrome among patients with *Campylobacter* infection: a general practice research database study. J Infect Dis. 2006;194:95–7. PubMed http://dx.doi.org/10.1086/504294

4. Baker MG, Kvalsvig A, Zhang J, Lake R, Sears A, Wilson N. Declining Guillain-Barré syndrome after campylobacteriosis control, New Zealand, 1988–2010. Emerg Infect Dis. 2012;18:226–33. PubMed http://dx.doi.org/10.3201/eid1802.111126

5. Vally H, Hall G, Dyda A, Raupach J, Knope K, Combs B, et al. Epidemiology of Shiga toxin producing *Escherichia coli* in Australia, 2000–2010. BMC Public Health. 2012;12:63–71. PubMed http://dx.doi.org/10.1186/1471-2458-12-63

6. Haagsma JA, Siersema PD, De Wit NJ, Havelaar AH. Disease burden of post-infectious irritable bowel syndrome in the Netherlands. Epidemiol Infect. 2010;138:1650–6. PubMed http://dx.doi.org/10.1017/S0950268810000531

7. Poropatich KO, Walker CL, Black RE. Quantifying the association between *Campylobacter* infection and Guillain-Barré syndrome: a systematic review. J Health Popul Nutr. 2010;28:545–52. PubMed http://dx.doi.org/10.3329/jhpn.v28i6.6602

8. Walker CL, Applegate JA, Black RE. Haemolytic-uraemic syndrome as a sequela of diarrhoeal disease. J Health Popul Nutr. 2012;30:257–61. PubMed http://dx.doi.org/10.3329/jhpn.v30i3.12288

9. Schwille-Kiuntke J, Frick JS, Zanger P, Enck P. Post-infectious irritable bowel syndrome—a review of the literature. Z Gastroenterol. 2011;49:997–1003. PubMed http://dx.doi.org/10.1055/s-0031-1281581

10. Hannu T. Reactive arthritis. Best Pract Res Clin Rheumatol. 2011;25:347–57. PubMed http://dx.doi.org/10.1016/j.berh.2011.01.018
